# Pharmacogenomics in practice: a review and implementation guide

**DOI:** 10.3389/fphar.2023.1189976

**Published:** 2023-05-18

**Authors:** Danya Kabbani, Reem Akika, Ahmed Wahid, Ann K. Daly, Ingolf Cascorbi, Nathalie Khoueiry Zgheib

**Affiliations:** ^1^ Department of Pharmacology and Toxicology, Faculty of Medicine, American University of Beirut, Beirut, Lebanon; ^2^ Department of Pharmaceutical Biochemistry, Faculty of Pharmacy, Alexandria University, Alexandria, Egypt; ^3^ Translational and Clinical Research Institute, Medical School, Newcastle University, Newcastle upon Tyne, United Kingdom; ^4^ Institute of Experimental and Clinical Pharmacology, University Hospital Schleswig-Holstein, Kiel, Germany

**Keywords:** guidelines, implementation, pharmacogenomics, practice, pharmacogenetics

## Abstract

Considerable efforts have been exerted to implement Pharmacogenomics (PGx), the study of interindividual variations in DNA sequence related to drug response, into routine clinical practice. In this article, we first briefly describe PGx and its role in improving treatment outcomes. We then propose an approach to initiate clinical PGx in the hospital setting. One should first evaluate the available PGx evidence, review the most relevant drugs, and narrow down to the most actionable drug-gene pairs and related variant alleles. This is done based on data curated and evaluated by experts such as the pharmacogenomics knowledge implementation (PharmGKB) and the Clinical Pharmacogenetics Implementation Consortium (CPIC), as well as drug regulatory authorities such as the US Food and Drug Administration (FDA) and European Medicinal Agency (EMA). The next step is to differentiate reactive point of care from preemptive testing and decide on the genotyping strategy being a candidate or panel testing, each of which has its pros and cons, then work out the best way to interpret and report PGx test results with the option of integration into electronic health records and clinical decision support systems. After test authorization or testing requirements by the government or drug regulators, putting the plan into action involves several stakeholders, with the hospital leadership supporting the process and communicating with payers, the pharmacy and therapeutics committee leading the process in collaboration with the hospital laboratory and information technology department, and healthcare providers (HCPs) ordering the test, understanding the results, making the appropriate therapeutic decisions, and explaining them to the patient. We conclude by recommending some strategies to further advance the implementation of PGx in practice, such as the need to educate HCPs and patients, and to push for more tests’ reimbursement. We also guide the reader to available PGx resources and examples of PGx implementation programs and initiatives.

## 1 Introduction

Interindividual variability in drug response is driven by several extrinsic and intrinsic factors, with genetic variations being increasingly recognized among these factors that lead to changes in the activity or availability of drug metabolizing enzymes (DMEs), receptors, channels, and other proteins involved in drug pharmacokinetics (PK) and pharmacodynamics (PD) (Thummel and Lin, 2014). Consequently, the term Pharmacogenomics (PGx), the study of interindividual variations in DNA sequence related to drug efficacy and toxicity, was coined. In this sense, PGx has become an effective tool to fulfill the promise of personalized medicine, while allowing patients to be treated based on their genetic makeup (Mitri et al., 2010; Bartlett et al., 2012).

Despite all emerging evidence and efforts enabling PGx, its clinical implementation has been suboptimal worldwide, especially in the developing world (Abou Diwan et al., 2019; Zgheib et al., 2020; El Shamieh and Zgheib, 2022). For instance, and in addition to global challenges such as the perceived lack of clinical utility and worries of disrupting the usual clinical pathways, other barriers may be attributed to local circumstances such as absence of national regulations for PGx testing, suboptimal infrastructure for the PGx integration into healthcare providers’ (HCP) workflow, lagging insurance plans for coverage of PGx testing, and lack of resources including national PGx data, guidelines, and necessary funds (Caraballo et al., 2017; Rigter et al., 2020; Pirmohamed, 2023).

Considerable efforts have been exerted to implement PGx into routine clinical practice. These efforts relied upon studies providing robust evidence for the benefit of PGx-guided therapeutic strategies. For instance, it has been reported that approximately 91%–99% of patients have at least one genotype that is associated with PGx actionable drugs, and that these drugs constitute up to 18% of all prescribed medications (Krebs and Milani, 2019). Moreover, a recently published study from the European Ubiquitous Pharmacogenomics (U-PGx) clinical implementation project showed that patients with PGx actionable test results, when treated according to Royal Dutch Association for the Advancement of Pharmacy - Pharmacogenetics Working Group (DPWG) recommendations, resulted in a lower percentage (21%) of clinically relevant adverse drug reactions (ADRs) compared to the control group (27.7%) that received standard treatment, though this difference was also seen for patients in the case group receiving nonactionable drugs (Swen et al., 2023). Further evaluations can be pursued in this study to address the influence of several genes, specific adverse reactions related to individual drugs, and phenoconversion caused by polypharmacy (Penas, 2023). In addition, it has been shown that ADRs and hospitalization resulting from drug toxicity can be better controlled by applying PGx. Cost savings from PGx-guided therapy can reach up to 3962 USD per patient per year even when test costs are considered (Luzum et al., 2021). More specifically, a systematic review that evaluated PGx-guided treatment of antidepressants and antipsychotic medications showed that 50% and 39% of the included studies revealed cost-effectiveness and cost-saving of PGx testing, respectively (Karamperis et al., 2021).

Considering the extensive evidence on the benefits of PGx and the availability of a myriad of resources enabling its clinical implementation, herein we propose an approach for initiating clinical PGx in the hospital setting, while acknowledging that implementation depends on local circumstances such as resources available, differences in insurance plans, and peculiarities of the health service’s organization, etc .,… To begin with, we introduce the stakeholders engaged in the implementation, evaluation, and improvement of the program. Next, we propose steps to be followed for developing and applying PGx in hospital clinical practice. We then discuss strategies to address the PGx awareness and training needs of HCPs and patients, and elaborate on the necessity of test reimbursement and how it can be enhanced. We also guide the reader to available PGx resources, and examples of PGx implementation programs and initiatives.

## 2 Stakeholders engaged in the clinical pgx design and implementation process

**FIGURE 1:**
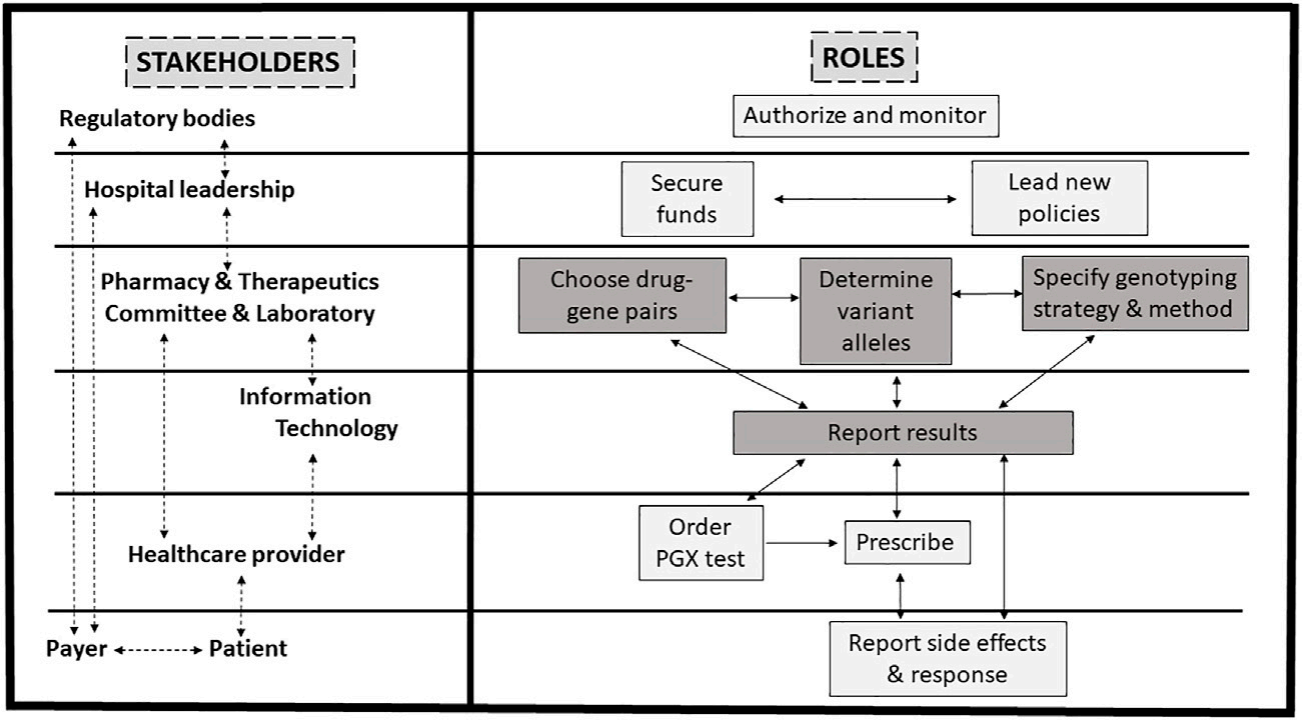
Proposed framework for pharmacogenomics (PGx) implementation in practice. PGx implementation in practice involves several stakeholders. See Box 1 for details. Briefly, after test authorization or requirements for testing by drug regulators, the hospital leadership supports the process and communicates with payers, while the pharmacy and therapeutics committee leads the process in collaboration with the hospital laboratory and information technology department. Healthcare providers order the test, make the appropriate therapeutic decisions, and explain them to the patient whom reports to the healthcare provider and payer. Steps for the design of the clinical PGx are highlighted in grey and detailed in Figure 2.

At least eight main stakeholders are involved in the PGx design and implementation process in the hospital setting (Figure 1; Box 1). These include drug regulators authorizing or requiring specific PGx tests, hospital leadership supporting the process and communicating with payers, pharmacy and therapeutics (P&T) committee leading the process in collaboration with the hospital, molecular laboratory and information technology (IT), and HCPs ordering the test, understanding the results, making the appropriate therapeutic decisions, and explaining them to the patients.

**BOX 1:** List and role of main stakeholders involved in the pharmacogenomics (PGx) implementation process in a hospital setting

Stakeholder	Role
**Regulatory bodies**	Authorize or require specific PGx tests.
	Provide guidelines and/or drug labeling.
	Monitor implementation.
	Communicate with hospital leadership and payers.
**Hospital leadership**	Secure funds and infrastructure.
	Lead the process and identify early adopters of change.
	Ensure compliance with ethical, legal and social issues.
	Monitor and evaluate the program’s impact.
	Contribute to and lead new policies.
	Communicate with regulatory bodies, payers, and pharmacy and therapeutics committee.
**Pharmacy & Therapeutics Committee**	Evaluate the available evidence in consultation with PGx consortia and networks.
	Review the most relevant drugs.
	Narrow down to the most actionable drug-gene pairs with related variant alleles.
	Monitor and evaluate the program for improvement.
	Communicate with hospital leadership, laboratory, information technology and healthcare providers.
**Laboratory**	Perform genotyping.
	Apply reactive point of care or preemptive testing.
	Choose candidate vs. panel testing.
	Communicate with pharmacy and therapeutics committee, information technology and healthcare providers.
**Information Technology**	Report and integrate results into electronic health records.
	Design clinical decision support systems.
	Communicate with pharmacy and therapeutics committee, laboratory, and healthcare providers.
**Healthcare provider**	Order the test.
	Understand and interpret the test results.
	Make therapeutic decisions.
	Educate the community.
	Communicate with patients, pharmacy and therapeutics committee, laboratory, and information technology.
**Patient**	Provide feedback on drug outcome
	Communicate with payers and healthcare providers.
**Payer**	Reimburse (or not) the test partially or fully
	Communicate with regulatory bodies, hospital leadership and patients.

### 2.1 Regulatory bodies

The FDA and EMA regulatory bodies in the US and Europe, respectively, are responsible for regulating the addition of PGx information and assessing the level of PGx labeling, be it required, recommended, actionable or informative (Ehmann et al., 2014; Mehta et al., 2020). Their recommendations are available through PharmGKB website, where HCPs can access all corresponding prescribing information and recommendations. In addition, guidelines on PGx data usage in drug development and labeling were established by the FDA and EMA. According to the FDA (United States Food and Drug Administration, 2005), the sponser may choose to submit with an investigational or marketing application PGx data that have not yet reached the status of a valid biomarker to, for example, correlate specific toxicities with genetic data, or inform the design of clinical trials. However, when the PGx data are known to affect safety in animals or efficacy or safety in humans, it is recommended that such data are submitted with the application. As for labeling, the PGx data may be included in an informational or actionable manner. Concerning the EMA (European Medicines Agency, 2010), and in the case of co-development of PGx biomarkers or assays, guidelines are put in place while reflecting on key scientific principles that need to be met to ensure compliance with good laboratory standards resulting in optimal reliability of the PGx assay.

Moreover, in the USA, the National Human Genome Research Institute encourages research conducted on health benefits and cost-effectiveness of genetic testing to promote genomic medicine (Vozikis et al., 2016). It also supports payers to enable reimbursement of genetic tests. On the other hand, in Europe, there are national regulatory bodies for each country, such as the Gemeinsamer Bundesausschuss in Germany, the Medicines and Healthcare Regulatory Agency in the United Kingdom, and La Haute Autorité de Santé in France (Vozikis et al., 2016). Depending on specific national regulations, they are responsible for authorizing, marketing, and/or monitoring the quality and safety of medicinal products. Their role is tuned by the government to ensure cooperation between various stakeholders -regulatory authorities, medical device manufacturers, payer organizations, academic/research institutes, wholesalers, laboratories, pharmaceutical companies, and HCPs-to ensure availability and affordability of medical supplies including genetic tests. Tests that were shown to improve healthcare were proposed to be included in a “positive medical device” list to enforce its use and reimbursement by public and private insurance companies (Vozikis et al., 2016).

### 2.2 Hospital leadership

The leadership group is the initial sponsor of the program. It is responsible for securing funds and infrastructure, ensuring compliance with ethical legal social issues (ELSI), and monitoring and evaluating the program’s impact. The leadership is fully engaged in the whole process and should be sensitive to the hospital culture and climate, including the readiness for change. It should identify early adopters of change or implementation champions (Tuteja et al., 2022). It may also present evidence to national officials to suggest amending regulations in favor of promoting the practice of personalized medicine, and developing reimbursement policies and educational programs (Hartzler et al., 2013; Cicali et al., 2022).

### 2.3 Developers: pharmacy and therapeutics committee, laboratory and information technology

Then comes the role of the program developers being the P&T committee in collaboration with the hospital’s laboratory and IT department. The P&T is a multidisciplinary committee that is responsible for all matters related to the use of medications in the institution, including the development and maintenance of the hospital formulary. For the sake of the proposed PGx program, we suggest the P&T committee, while in constant communication with expert PGx consortia and networks, to be responsible for evaluating the available PGx evidence, reviewing the most relevant drugs, and narrowing down to the most actionable drug-gene pairs with related variant alleles to be tested. P&T members also discuss and decide with laboratory experts whether to apply reactive point of care or preemptive testing and on the genotyping strategy being a candidate or panel testing. The P&T committee also collaborates with IT to find the best way to report and interpret PGx test results with the option of integration into electronic health records (EHRs) coupled with clinical decision support (CDS) systems. It follows the program’s progress for improvement (Hartzler et al., 2013; Cicali et al., 2022).

### 2.4 Users: healthcare providers and patients

After that comes the role of the program users, being HCPs and patients. HCPs order the PGx test paired with the drug they plan to prescribe for a specific therapeutic need, interpret test results, communicate with patients, prescribe the personalized dose, or choose an alternative medication as applicable. HCPs also have an essential role in educating the community and patients on PGx and how it can impact their treatment. They can help monitor the general attitude toward PGx implementation and propose strategies to increase PGx awareness. These include educational workshops and conferences, TV and social media talks, and billboard and brochure advertisements that introduce the program to the public. HCPs also must provide feedback to the P&T, the molecular laboratory, and IT personnel regarding the process of including CDS in order to enhance the efficiency and efficacy of the implemented system (Hartzler et al., 2013; Cicali et al., 2022). Patients’ attitudes towards the PGx testing should also be taken into consideration. They should be informed about regulations that protect them from genetic discrimination by insurance companies and employers. They also should be informed regarding reimbursement policies and whether testing is entirely, partially, or not covered (Hartzler et al., 2013; Cicali et al., 2022).

### 2.5 Payers

Finally comes the role of payers, which may be public or private health insurance plans, research grants, laboratory reimbursement plans, out-of-pocket, or others. Although many potential payers are still reluctant to reimburse the PGx implementation or test, the growing evidence on the clinical utility of PGx testing is pushing toward fulfilling the right of patients to receive individualized treatment and to be protected by public policies and regulations that are integrated into national public or private health plans (Tuteja et al., 2022). Establishing or updating well-defined regulations will ultimately force insurance companies to revise their coverage plans to enable PGx testing. A success story is the experience of genotyping for *DPYD* variant alleles upon fluoropyrimidines prescribing whereby the resulting clinical and economic benefits led to securing governmental financial support in Ontario, with further evidence of cost-effectiveness probably leading to expansion of the experience to other medical institutions (Brooks et al., 2022; Medwid and Kim, 2022; Varughese et al., 2022). Moreover, the EMA recommended testing for DPYD, but concerns regarding the economic benefit and cost-effectiveness of testing resulted in slow adoption of the recommendation. Thus, studies were initiated in some countries to address cost-effectiveness and potential for improvement of quality of life as a result of DPYD genotyping prior to fluoropyrimidine-based chemotherapy (Deenen et al., 2016; Brooks et al., 2022). One study showed that DPYD screening is a cost-effective strategy and improves survival by 0.0038 quality adjusted life years (Brooks et al., 2022). Another study showed that genotype-guided dosing reduces grade 3 and above toxicity from 73% to 28%, drug-induced death from 10% to 0%, and average treatment cost per patient (Deenen et al., 2016). The EMA recommendation resulted in a requirement for DPYD genotyping in the UK (Tsiachristas et al., 2022). Also, guidelines for DPYD testing have been issued in other European countries such as The Netherlands, Italy, Germany and France (Tsiachristas et al., 2022) with a mandatory character.

## 3 Design of clinical pgx in the hospital setting

**FIGURE 2:**
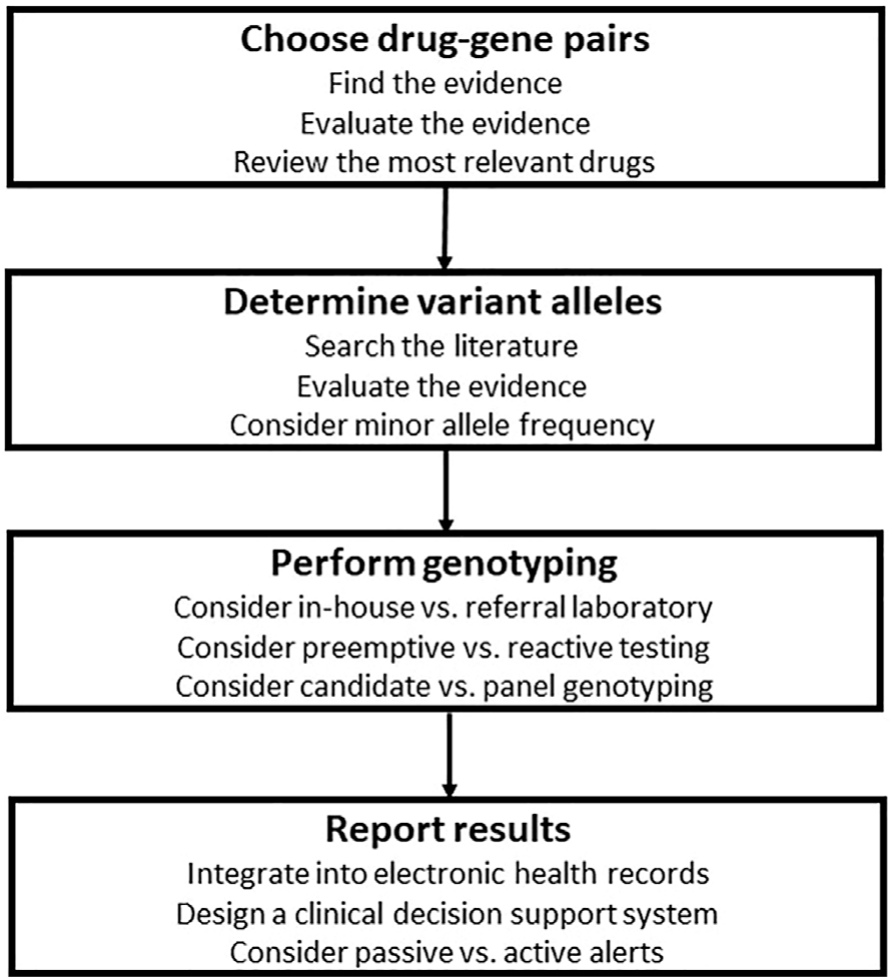
Steps for the design of clinical pharmacogenomics (PGx) in the hospital setting. The first step is to evaluate the available PGx evidence, review the most relevant drugs, and narrow down to the most actionable drug-gene pairs, then is to choose variant alleles based on population specific minor allele frequencies and data curated and evaluated by experts and drug regulatory authorities. Next is to decide with the molecular laboratory on the genotyping strategies and methods. Then is to work out with the information technology team the best way to interpret and report PGx test results, ideally into patients’ electronic health records if available, and with clinical decision support systems if feasible.

As shown in Figures 1, 2, designing a clinical PGx program in the hospital setting entails several steps. The P&T committee should evaluate the available PGx evidence, review the most relevant drugs, and narrow down to the most actionable drug-gene pairs and related variant alleles based on data curated and evaluated by experts and drug regulatory authorities. The next step is to decide with the molecular laboratory on the genotyping strategies and methods. Then is to work out with the IT team the best way to interpret and report PGx test results, ideally into patients’ EHRs if available, and with CDS systems if feasible.

### 3.1 Choosing the top drug-gene pairs

In order to choose the top drug-gene pairs to be initially implemented, the P&T committee should first consult external expert sources such as PGx consortia and networks to find and evaluate the most substantial evidence for PGx testing. Then, the list of drugs and related genes can be narrowed down based on the reviewed evidence.

#### 3.1.1 Finding the evidence

The implementation of PGx programs requires high-quality and consistent evidence that can be translated into regulations and guidelines (Luzum et al., 2021). These can be compiled from available PGx resources such as consortia, networks, societies, and regulatory agencies.

Several consortia and networks, some of which are listed and described in Table 1, were launched in the attempt to increase awareness, facilitate adoption, and provide the guidance necessary for integration of PGx programs into clinical practice. The Pharmacogenomics Global Research Network (PGRN) (Pharmacogenomics Research Network PGRN, 1998) is one of the first professional communities to work on PGx implementation. It has been heading several projects to include the recruitment and genotyping of people as part of a research protocol for the evaluation of the utility of the PGx endeavor on drug response. In addition, and as part of the Electronic Medical Records and Genomics Network (e-MERGE) (Electronic Medical Records and Genomics Network e-MERGE, 2007), the PGRN has been working on and proposing ways to upgrade EHR systems to be compatible with genetic results storage, as well as designing CDS for drug-gene pairs to guide HCPs in test ordering, interpretation, and drug prescription. Similarly, the Implementing Genomics in Practice (IGNITE) (Implementing Genomics in Practice IGNITE, 2013) network provides guidance for genomic implementation in healthcare, and provides a guiding toolbox for clinicians. Moving forward, professional PGx communities and programs progressed to provide improved PGx implementation models, clinical utility evidence, and comprehensive resources. All these efforts produced a set of valuable databases and tools that allow getting information on the drug and genes affecting its response, such as with the Pharmacogenomics Knowledge Base (PharmGKB) (Pharmacogenomics Knowledge Base PharmGKB, 2001; Whirl-Carrillo et al., 2012; Whirl-Carrillo et al., 2021), the genes, variants, frequencies and their phenotype with the Pharmacogene Variation Consortium (PharmVar) (Pharmacogene Variation Consortium PharmVar, 2000; Gaedigk et al., 2018; Gaedigk et al., 2020; Gaedigk et al., 2021), recommendations on what genetic variants should be tested to get interpretable results by the Association of Molecular Pathology (AMP) (Association of Molecular Pathology AMP, 1995), templates for creating genotyping result reports by the Pharmacogenomics Clinical Annotation Tool (PharmCat) (Sangkuhl et al., 2020; Pharmacogenomics Clinical Annotation Tool PharmCat, 2022), and recommendations on what to do when a PGx drug is prescribed by the Clinical Pharmacogenetics Implementation Consortium (CPIC) (Clinical pharmacogenetics implemetation Consortium CPIC, 2009).

In addition to the above-described CPIC, few other professional societies have established guidelines for PGx practice (Pharmacogenomics Knowledge Base PharmGKB, 2017), including the DPWG (The Dutch Pharmacogenetics Working Group DPWG, 2005), the Canadian Pharmacogenomics Network for Drug Safety (CPNDS) (Canadian Pharmacogenomics Network for Drug Safety CPNDS, 2004) and the French National Network of Pharmacogenetics (RNPGx) (Picard et al., 2017), among others. In addition, drug regulatory agencies (Pharmacogenomics Knowledge Base PharmGKB, 2017) including the EMA (European Medicinal Agency EMA, 1995) and the US FDA, have incorporated PGx information and prescribing tags in the approved drug labels, with the FDA allocating a specific and regularly updated link to all approved drugs with PGx label annotations (Mehta et al., 2020; United States Food and Drug Administration, 2022a).

#### 3.1.2 Evaluating the evidence

The P&T may choose to build on guidelines or regulations established in one’s country if available. For many countries, however, such regulations are not available, institutions would hence have to compare and contrast various resources and choose what is most applicable to their local context. The Office of Public Health Genomics at the US Center for Disease Control and Prevention (CDC) proposes a model for the evaluation and integration of genomic tests based on four components, A, C, C, and E, with A being analytical validity that addresses the accuracy and reliability of genetic testing, C being clinical validity that looks at the accuracy and reliability by which the test predicts the associated drug outcome, C being the clinical utility as the risks and benefits resulting from introducing genetic tests into clinical practice on the community, and E for ELSI being the associated ethical and regulatory policies (Centers for Disease Control and Prevention, 2000).

Similar frameworks have been applied by the FDA and EMA regulatory authorities and the PharmGKB to come up with evidence-based annotation levels, and the CPIC for their implementation guidelines. All provide recommendations on whether to use a drug, adjust the dose or switch to an alternative based on rigorously evaluated evidence. The PharmGKB curates and analyzes available studies to provide annotations for drugs and gene variants, while assigning a level of evidence based on an elaborate scoring system that depends on two main factors. First, the variant annotation score is calculated by a stepwise process that considers all aspects of the evaluated studies including phenotype category, *p*-value, cohort and effect size, study type and the presence of a significant association. Second is the presence of a clinical guideline and or a drug label. Level of evidence for drug-gene variants ranges from 1 to 4, with 1A being supported by solid and non-conflicting data, while pairs assigned a level of evidence of 4 lack supporting data (Whirl-Carrillo et al., 2012; Whirl-Carrillo et al., 2021). As for CPIC, A, B, C, and D levels are designated such that A and B imply that evidence favors changing the drug prescription to a genetically safer one. In contrast, C and D imply that evidence did not reach a level to suggest a genetically-based prescription. CPIC also applies a framework to rank its recommendations as strong, moderate, or optional based on supporting studies such as randomized clinical trials (RCTs) and *in vivo* PK/PD studies (Caudle et al., 2016).

#### 3.1.3 Narrowing down to a list of top drug-gene pairs

We propose that institutions that do not have country-specific guidelines or regulations compile all available PGx data from the PharmGKB, CPIC, FDA, and EMA. See Supplementary Table S1 as an example. We tabulated all drugs that are either listed in the PharmGKB’s Drug Label Annotations table under FDA or the Clinical Guideline Annotations table under CPIC. We also added the PharmGKB level of evidence for clinical annotations and all CPIC recommendations, and FDA and EMA PGx levels and drug labels when available. Of note that some discordance can be noted among the various resources despite being derived from the same evidence base (Koutsilieri et al., 2020; Shekhani et al., 2020; United States Food and Drug Administration, 2022b; Pirmohamed, 2023). For instance, many of the drugs labeled as “testing required” by the FDA are not mentioned in EMA labels. Moreover, many drug-gene associations listed by the FDA are neither listed by EMA nor by CPIC (See Supplementary Table S1). More specifically for clopidogrel prescription, for example, CPIC (Scott et al., 2013) and FDA (United States Food and Drug Administration, 1997) recommend the use -or consideration of the use-of alternative drugs in CYP2C19 poor metabolizers. At the same time, the EMA label (European Medicines Agency, 1998) does not make such a specific recommendation. Hence the role of the P&T committee to assess these inconsistencies and to make an informed decision on what model to follow and what drugs to include.

Two methods can be applied concomitantly or independently to narrow down the list of drug-gene pairs to be initially implemented. First, one can evaluate and choose the most frequently prescribed drugs in one’s setting. For example, a program initiated in Africa should include drugs like chloroquine, HIV-protease inhibitors, and isoniazid used to treat malaria, HIV, and tuberculosis, respectively (Greenwood, 2004; Marais et al., 2013; Dandara et al., 2019), while recognizing that non-communicable diseases are also an important cause of morbidity and mortality in developing countries (Grant and De Cock, 1998; Kennedy et al., 2007; Dean et al., 2020). If such data are unavailable, one can refer to the World Health Organization (WHO) list of essential drugs (World Health Organization WHO, 2021). The second method is to review available literature in other institutions or countries on the most commonly prescribed drugs supported by solid evidence of clinical utility for PGx. Several studies were conducted on multiple populations (Schildcrout et al., 2012; Samwald et al., 2016; Caraballo et al., 2017; Chanfreau-Coffinier et al., 2019; Hicks et al., 2021). Samwald et al. (2016) classified the most frequently used PGx drugs in the USA within different age groups based on a model whereby PGx drug exposure data were collected from insurance databases, the drugs that had CPIC or DWPG guidelines were then highlighted, followed by the selection of the most frequently used drugs, while considering different ethnic groups. This analysis showed that opioids (codeine, oxycodone, and please inset here: groups. This analysis showed that opioids (codeine, oxycodone, and tramadol) were primarily used in the younger population, while cardiovascular drugs (simvastatin, clopidogrel, and warfarin) were frequently prescribed for older people (Samwald et al., 2016; Hicks et al., 2021). Similar results appeared from a survey conducted by IGNITE group with 11 healthcare systems, whereby each site provided the available e-prescription records of adults above 18 years for in and outpatient settings (Hicks et al., 2021). A third study conducted at Vanderbilt University Medical Center reached a similar conclusion, adding some oncology drugs to the top 25 PGx most prescribed drugs (Schildcrout et al., 2012). In this study, drugs having FDA PGx prescribing information were considered. Consequently, a list of drugs was suggested by several investigators as ready to be implemented. This list includes but is not limited to: statins, clopidogrel, and warfarin for cardiovascular diseases, codeine and tramadol as opioid analgesics, ondansetron and proton pump inhibitors for gastrointestinal illnesses, fluoropyrimidines, tamoxifen, and thiopurines for cancer, and antidepressants such as the selective serotonin reuptake inhibitors (Weitzel et al., 2019; Rollinson et al., 2020; Medwid and Kim, 2022). Finally, a fourth study evaluated the longitudinal exposure in primary care in the United Kingdom of a list of 63 drugs identified from the PharmGKB database to be associated with 19 pharmacogenes. The authors showed that most of the prescribed PGx drugs were for pain relief, gastrointestinal protection, and psychiatric and cardiovascular conditions, and that more than 95% of these drugs are affected by three pharmacogenes: CYP2C19, CYP2D6, and SLCO1B1 (Kimpton et al., 2019).

Based on these two methods, we narrowed down the list of 499 drugs from Supplementary Table S1 into 59 drugs with related pharmacogenes (Table 2). We limited the list to drugs associated with germline non-somatic PGx tests. As such, we did not include genes for targeted anticancer drugs or immunotherapy. We also checked the Tier 1 Very Important Pharmacogenes (VIP) list from PharmGKB (Pharmacogenomics Knowledge Base PharmGKB, 2020), and kept some drug-gene pairs such as TPMT and NUDT15 for thioguanine despite being non-level 1A per PharmGKB level of evidence. We chose so for the mere fact that both VIP are also associated with more substantial evidence with other drugs such as mercaptopurine and azathioprine. We excluded VIP for drugs not commonly used in the community worldwide such as CFTR, F5, and RYR1 related to cystic fibrosis treatment, thrombopoeitin receptor agonists, and anesthetic drugs, respectively. We also did not include NAT2 for isoniazid as the drug-gene pair has so far not been addressed by the CPIC.

**TABLE 1:** Few programs and resources for the implementation of Pharmacogenomics (PGx).

Program or resource with website link	Description
**AMP:** Association of Molecular Pathology https://www.amp.org/	➢ It is an international non-profit scientific society that aims to enhance the science and clinical practice of molecular and genomic laboratories
	➢ It provides guidelines and global expertise in the field of molecular pathology
	➢ It also provides recommendations for the choice of genetic variants that ought to be tested
**CPIC:** Clinical Pharmacogenetics Implementation Consortium https://cpicpgx.org/	➢ It is an international consortium of volunteers and staff that aim to facilitate the use of PGx tests in clinical care
	➢ It creates, curates, and posts freely available, peer-reviewed, evidence-based, updatable, and detailed gene/drug clinical practice guidelines
	➢ All guidelines are published in the Clinical Pharmacology and Therapeutics journal
**e-MERGE:** Electronic Medical Records and Genomics Network https://emerge-network.org/	➢ It is a US network of academic medical centers that integrate genomic data with EHR
	➢ It aims to sequence and clinically implement relevant genotypes into healthcare through EHR and CDS incorporation. It also aims to discover and assign phenotypes of rare and presumably clinically relevant variants
	➢ It provides resources and tools (informatics, education) including EHR and CDS infrastructure to assist in the implementation of PGx into practice. ​CDS-KB (Clinical Decision Support Knowledgebase) (https://cdskb.org/) is one of the tools that is supported by *e-MERGE* in collaboration with *IGNITE* shown below
**IGNITE:** Implementing Genomics in Practice https://gmkb.org/ignite-gdp/	➢ It is a US network that supports genomic implementation in healthcare setting
	➢ It aims to develop, use, and evaluate new strategies and clinical models for implementing individuals’ genomic information into clinical practice
	➢ It provides a Toolbox ​ for clinicians that consists of a collection of genomic practice models related to disease diagnosis, pharmacogenomics and risk assessment. And for researchers, it provides guides and educational material on data collection, laboratory testing, research and training development tools. It has also developed a map for reimbursement of PGx tests
**PGRN:** Pharmacogenomics Global Research Network https://www.pgrn.org/what-is-pgrn.html	➢ It is a community driven international network that includes academic institutions, diagnostic laboratories, biotechnology, pharmaceutical industry, and clinical practitioners
	➢ It aims to guide and lead precision medicine for actionable variants, and to establish a worldwide collaboration of PGx researchers with a focus on supporting PGx in developing countries
	➢ It provides its members with links to implementation resources, algorithms for PGx-based dosing, PGx competencies for teachers, Research-in-Progress Seminar series (RIPS), and patient education
**PharmCAT:** Pharmacogenomics Clinical Annotation Tool https://pharmcat.org/	➢ It is a software tool that can extract CPIC PGx variants and represent them with the suitable star allele haplotype/diplotype
	➢ It provides interpretation, and generates a report for the variant alleles
**PharmGKB:** Pharmacogenomics Knowledge Base https://www.pharmgkb.org/	➢ It is a publicly available resource that is responsible for the integration and dissemination of information related to genomic variation and drug response
	➢ It aims to help healthcare providers and researchers find information about genetic polymorphisms and their effect on drugs’ efficacy and safety
	➢ The website includes information and links to curated pathways, Very Important Pharmacogenes (VIP), PGx prescribing information, drug label PGx annotations, as well as PGx variant and clinical annotations based on updated evidence-based criteria
**PharmVar:** Pharmacogene Variation Consortium https://www.pharmvar.org/	➢ It is a central repository for PGx haplotypes and allelic variants with a focus on drug metabolizing enzymes
	➢ It aims to facilitate basic and clinical research and the interpretation of PGx tests’ results
	➢ It also provides a unifying designation system (nomenclature) for the global PGx community

EHR: electronic health records; CDS: clinical decision support.

### 3.2 Choosing the variants to be tested

After determining the drug-gene pairs, the P&T committee should specify the variants that should be tested in coordination with Laboratory experts and personnel. We describe in Table 3 a list of variants associated with the genes proposed in Table 2. This list is non-exhaustive and laid out for illustrative purposes only. It is based on the most commonly proposed variants and genes in the literature (Samwald et al., 2016; van der Wouden et al., 2017; Chanfreau-Coffinier et al., 2019; van der Wouden et al., 2019; Rollinson et al., 2020; Medwid and Kim, 2022) and supported with high quality evidence, clinical guidelines and/or drug labels with genetic information. We also chose variants that are relatively common in Africans, Asians or Europeans. Each genetic variant has a unique identifier (rsID) as per the NCBI dbSNP database (National Center for Biotechnology Information NCBI, 1999). The *allele nomenclature and genotype-phenotype relation can be extracted from the PharmGKB and/or PharmVar resources.

**TABLE 2:** Proposed drug-gene pairs for the clinical implementation of pharmacogenomics (PGx).

Indications		Drugs		Genes	PHARMGKB^b^ https://www.pharmgkb.org/	CPIC^c^ https://cpicPGx.org/		FDA^d^ https://www.fda.gov/		EMA^e^ https://www.ema.europa.eu/en	
	Classes	Name	On WHO list of essential medicines^a^		Level of evidence of PGx clinical annotation	Recommendation	Drug label annotation Tag	PGx level	Drug label annotation Tag	PGx level	Drug label annotation Tag
Autoimmune Diseases	Antigout	Allopurinol	Yes	HLA-B	1A	Yes	Pediatric	Testing Recommended	Alternate drug, Prescribing info	-	-
Cardiovascular Diseases	Anticoagulant	Warfarin	Yes	CYP2C9, VKORC1	1A	Yes	Pediatric	Actionable PGx	Prescribing Info	-	-
				CYP4F2	1A	Yes	Pediatric	-	-	-	-
	Antiplatelet	Clopidogrel	Yes	CYP2C19	1A	Yes	Pediatric	Actionable PGx	Prescribing Info	Actionable PGx	-
	Statins	Atorvastatin	Yes	SLCO1B1	1A	Yes	Dosing info, Pediatric	Informative PGx	-	-	-
		Rosuvastatin	-	ABCG2, SLCO1B1	1A	Yes	Alternate drug, Dosing info, Pediatric	Actionable PGx	-	-	-
		Simvastatin	Yes	SLCO1B1	1A	Yes	Alternate drug, Dosing info, Pediatric	Informative PGx	-	-	-
Gastrointestinal diseases	Antiemetic	Ondansetron	Yes	CYP2D6	1A	Yes	Pediatric	Informative PGx	-	-	-
	Proton pump inhibitors	Dexlansoprazole	-	CYP2C19	1A	Yes	Pediatric	Actionable PGx	-	-	-
		Lansoprazole	-	CYP2C19	1A	Yes	Pediatric	Informative PGx	-	-	-
		Omeprazole	Yes	CYP2C19	1A	Yes	Pediatric	Actionable PGx	-	-	-
		Pantoprazole	-	CYP2C19	1A	Yes	Pediatric	Actionable PGx	Prescribing info, Pediatric	-	-
Infectious diseases	Antibiotics	Gentamicin	Yes	MT-RNR1	1A	Yes	Alternate drug, Pediatric	-	-	-	-
		Streptomycin	-	MT-RNR1	1A	Yes	Alternate drug, Pediatric	-	-	-	-
		Tobramycin	Yes	MT-RNR1	1A	Yes	Alternate drug, Pediatric	-	-	-	-
	Antifungal	Voriconazole	Yes	CYP2C19	1A	Yes	Pediatric	Actionable PGx	-	Informative PGx	-
	Antivirals	Abacavir	Yes	HLA-B	1A	Yes	Pediatric	Testing Required	Alternate drug, Prescribing info	Testing Required	Alternate drug, Prescribing info
		Atazanavir	Yes	UGT1A1	1A	Yes	Pediatric	-	-	-	-
		Efavirenz	Yes	CYP2B6	1A	Yes	Pediatric	Actionable PGx	-	Actionable PGx	-
Neuropsychiatric diseases	Antiepileptics	Carbamazepine	Yes	HLA-A	1A	Yes	Alternate drug, Pediatric	Actionable PGx	-	-	-
				HLA-B	1A	Yes	Alternate drug, Pediatric	Testing Required	Alternate drug, Prescribing info	-	-
		Oxcarbazepine	-	HLA-B	1A	Yes	Alternate drug	Testing Recommended	Alternate drug, Prescribing info	-	-
		Phenytoin	Yes	CYP2C9, HLA-B	1A	Yes	Pediatric	Actionable PGx	Prescribing info	-	-
	Non-opioid analgesic	Celecoxib	-	CYP2C9	1A	Yes	Pediatric	Actionable PGx	Dosing info, Prescribing info	-	-
	Opioid analgesics	Codeine	Yes	CYP2D6	1A	Yes	Pediatric	Actionable PGx	Alternate drug, Prescribing info, Pediatric	-	-
		Tramadol	-	CYP2D6	1A	Yes	Pediatric	Actionable PGx	Alternate drug, Prescribing info	-	-
	Antidepressants	Amitriptyline	Yes	CYP2C19	1A	Yes	Pediatric	-	-	-	-
				CYP2D6	-	Yes	Pediatric	Actionable PGx	-	-	-
		Aripiprazole	-	CYP2D6	1A	-	-	Actionable PGx	Dosing info, Prescribing info	Actionable PGx	Dosing info, Prescribing info
		Citalopram	Yes	CYP2C19	1A	Yes	Pediatric	Actionable PGx	Dosing info, Prescribing info	-	-
		Clomipramine	Yes	CYP2C19	1A	Yes	Pediatric	-	-	-	-
				CYP2D6	1A	Yes	Pediatric	Actionable PGx	-	-	-
		Desipramine	-	CYP2D6	1A	Yes	Pediatric	Actionable PGx	-	-	-
		Doxepin	-	CYP2C19, CYP2D6	1A	Yes	Pediatric	Actionable PGx	-	-	-
		Escitalopram	Yes	CYP2C19	1A	Yes	Pediatric	Actionable PGx	-	-	-
		Fluvoxamine	Yes	CYP2D6	1A	Yes	Pediatric	Actionable PGx	-	-	-
		Imipramine	-	CYP2C19	1A	Yes	Pediatric	-	-	-	-
				CYP2D6	1A	Yes	Pediatric	Actionable PGx	-	-	-
		Nortriptyline	-	CYP2D6	1A	Yes	Pediatric	Actionable PGx	-	-	-
		Paroxetine	Yes	CYP2D6	1A	Yes	Pediatric	Informative PGx	-	-	-
		Sertraline	Yes	CYP2C19	1A	Yes	Pediatric	-	-	-	-
		Trimipramine	-	CYP2C19	1A	Yes	Pediatric	-	-	-	-
				CYP2D6	1A	Yes	Pediatric	Actionable PGx	-	-	-
		Venlafaxine	-	CYP2D6	1A	-	-	Actionable PGx	-	-	-
Oncology	Cytotoxic therapy	Capecitabine	Yes	DPYD	1A	Yes	Pediatric	Actionable PGx	Prescribing Info	Testing Recommended	Alternate drug, Prescribing info
		Fluorouracil	Yes	DPYD	1A	Yes	Pediatric	Actionable PGx	Alternate drug, Prescribing info	-	-
		Mercaptopurine	Yes	NUDT15, TPMT	1A	Yes	Pediatric	Testing Recommended	Dosing info, Prescribing info	Actionable PGx	Prescribing Info
		Thioguanine	-	NUDT15, TPMT	3	Yes	Pediatric	Testing Recommended	Dosing info, Prescribing info	-	-
	Hormonal therapy	Tamoxifen	Yes	CYP2D6	1A	Yes	-	Actionable PGx	-	-	-
	Immunosuppressants	Azathioprine	Yes	NUDT15, TPMT	1A	Yes	Pediatric	Testing Recommended	Prescribing info	-	-
		Tacrolimus	Yes	CYP3A5	1A	Yes	Pediatric	Informative PGx	-	-	-

a https://www.who.int/publications/i/item/WHO-MHP-HPS-EML-2021.02

b PharmGKB, clinical annotations levels range from 1-4, with level 1 meeting the highest criteria.

c CPIC, provides guidelines that help in implementing pharmacogenomics tests into clinical practice setting.

d FDA, approves and monitors drugs and other biological products for ensuring safety and effectiveness in the United States of America.

e EMA, approves and monitors drugs and other biological products for ensuring safety and effectiveness within the European union and economic area.

Testing Required: Genetic testing is obligatory before prescribing the drug.

Testing Recommended: Genetic testing should be considered before prescribing the drug.

Actionable PGx: Genetic testing is available and advised due to possible gene-drug relation, but it is not required.

Informative PGx: Genetic testing is not required. Gene variants do not affect drug response, or the effect is not clinically significant.

Dosing info: Tag implies that dose adjustment is required.

Alternate drug: Tag implies that a drug substitute is recommended.

Prescribing info: Tag implies that dose adjustment or drug substitution is either required or suggested, drug should be used with caution, or patients should be monitored for adverse reactions.

Pediatric: Tag implies that a drug contains PGx information for pediatric population.

The most crucial consideration for the choice of the target variants is the Minor Allele Frequency (MAF) of the local population or ethnicities (Van Driest et al., 2014). For instance, while in East Asians, HLA-B*15:02 allele is relatively common, and its PGx testing for carbamazepine is required to prevent Stevens–Johnson syndrome (SJS) and toxic epidermal necrolysis (TEN), the same test is not required in other populations where the allele is quite rare (United States Food and Drug Administration, 2009). Another example is mercaptopurine, whereby only TPMT genotyping is recommended in Caucasians, while both TPMT and NUDT15 genotyping should be performed for East Asians who may carry some common actionable NUDT15 variants (Yang et al., 2015). A third example comprises CYP2C9 genotyping test before prescribing warfarin to African Americans, whereby the test should include CYP2C9 *2, *3, *5, *6, *8, and *11 *versus* only *2 and *3 for Europeans (Johnson et al., 2017). Another important consideration to be kept in mind is how well the variants reflect the phenotype of the enzyme activity. For this purpose, the AMP guides on the basic variants that should be tested together to allow accurate interpretation of the gene’s phenotype. For example, at least the *2, *3, and *17 should be included when genotyping for CYP2C19, while other additional variants are optional. This classification was based on the prevalence of these variants in different ethnic groups and their functional effect on enzyme activity and drug response (Pratt et al., 2018). Of note that one has to constantly review the literature for emergent variants or haplotypes such as the common CYP2C:TG haplotype, defined by rs2860840T and rs11188059G co-occurrence, that is associated with CYP2C19 increased enzyme activity, hence affecting metabolism of drugs such as sertraline and escitalopram (Braten et al., 2021; Braten et al., 2022).

### 3.3 Specifying genotyping strategies and methods

Herein, two strategic decisions should be made by the P&T committee in coordination with the molecular laboratory. The first decision is whether to genotype for the selected drug-gene pairs preemptively or reactively. The second is whether to test for one or only a few candidate gene variants or perform more extensive panel genotyping. The points to be considered include, in addition to funds or reimbursement matters, availability of in-house or reference laboratories, IT expertise, EHR interfaces capable of holding and interpreting genetic test results, and well-trained HCPs able to deal with genetic data.

#### 3.3.1 Laboratory considerations

The medical institution may either establish, already have a certified genotyping laboratory, or refer to an outside reference laboratory to perform the PGx test. Such decisions mainly depend on availability of expertise, resources and funds as well as the extent of demand for PGx testing. The Genetic Testing Registry website (National Center for Biotechnology Information NCBI, 2012; Rubinstein et al., 2013) presents information on already available genetic and PGx tests in the United States. Also, the National genomic test directory (National genomic test directory, 2022) provides genomic tests commissioned by the National Health Services in the United Kingdom. One can search for a specific gene of interest and get a list of tests with details on purpose and coverage, validity, genotyping methodology, associated evidence for effectiveness, and contact laboratories with credentials.

Regardless of whether the lab is in-house or contracted, four primary standards must be considered (Vo et al., 2017). First, and as noted above, the pharmacogene(s)’ selection should be relevant to the tested population, and feasible with the available technology being a candidate or panel genotyping. Second, there must be documented evidence of good laboratory practices such as the College of American Pathologists accreditation and Clinical Laboratory Improvement Amendments certification for the USA, the European co-operation for Accreditation for Europe (European co-operation for Accreditation, 2022), and the International Laboratory Accreditation Cooperation for the international level (International Laboratory Accreditation Cooperation, 2022), to ensure accuracy, reproducibility, sensitivity, and specificity of the assay performed with reference and reportable ranges (Bristol, 2002; Endrullat et al., 2016). Third, the format of lab reports should be designed to include simple stand-alone gene results with or without interpretative comments. These are integrated into the EHR or an online portal, if available, or simple paper reports. Finally, it is important to negotiate with reference laboratories outside the institution on cost or reimbursement models with the possibility for financial assistance or partnerships as applicable (Tuteja et al., 2022). In the USA, genetic testing companies may facilitate the process through contracting payers to reimburse patients who are required to undergo the test, or making special discounts for out-of-pocket payers. In addition, the growing body of genetic testing companies increases the competition among them leading to better offers for the consumer (Wolff and Wolff, 2018). In Europe, pricing policies are developed to restrict manufacturers’ power in controlling genetic testing prices to ensure availability and protect consumers from exaggerated charges (Vozikis et al., 2016).

#### 3.3.2 Preemptive *versus* reactive testing

Genes are stable, and genetic data remain unchanged with time; hence it is a practical call to genotype high-risk gene variants and store them in EHRs before a PGx drug is needed. This preemptive genetic testing approach saves critical time and allows HCPs to prescribe PGx medications directly when required. This approach protects the patient from trial and error with unwanted ADRs (Roden et al., 2018). Nevertheless, this strategy comes at a higher cost, necessitates complex technologies and EHR integration, and is typically not reimbursed as payers may not see the value of the test at the time of the request (Keeling et al., 2019; Haidar et al., 2022a). The other approach, the reactive point of care testing, is to order the genotyping test when a PGx drug is to be prescribed. Although less costly, this approach may not be timely since a drug prescription cannot be postponed in many cases, and the patient may suffer ADRs due to the wrong dosage or drug choice until the results are out (Nicholson et al., 2021; Haidar et al., 2022a). It is up to the institution to decide on which strategy is most suitable depending on funding and feasibility in the local context.

#### 3.3.3 Candidate *versus* panel genotyping

As noted above, the medical institution may perform the planned PGx test in-house or refer to an outer reference laboratory. It should also strategize on choosing the candidate instead of panel genetic testing. Conversely, candidate genetic tests cover one or few specific gene variants related to a particular PK or PD pathway. The advantage of this type of test is that it is more accessible, less time-consuming, and less challenging to perform and interpret. It is mostly Polymerase Chain Reaction (PCR)-based or performed on small microarrays (Krebs and Milani, 2019; van der Lee et al., 2020; Verlouw et al., 2021). On the other hand, panel and genome-wide genotyping allow coverage of a more significant number of variants (Krebs and Milani, 2019; van der Lee et al., 2020; Verlouw et al., 2021). These tests may be ordered in patients taking multiple PGx actionable drugs or suspected to be prescribed various drugs based on age, comorbidities, or family history. This method is considered cost-effective since several genes can be genotyped together as one pool which decreases the cost per gene. For example, one may choose a panel of variants for drug transporters and CYP enzymes for a patient suffering from hypercholesterolemia to decide on the actionable rosuvastatin prescription. Since hypercholesterolemia is a risk factor for myocardial infarction, the panel can also cover variants for genes involved in the PK pathway of other cardiovascular drugs such as the antiplatelet clopidogrel. Panel tests are more challenging to deal with due to the large amounts of data they generate. In addition, this approach may not be practical in cases where the patient requires imminent treatment. Note that although panel testing includes many variants, one can decide to report only those associated with the prescribed drugs into the EHR. At the same time, the remaining results are stored in a separate database. This approach decreases the amount of data the HCP has to deal with, but at the same time, the data are readily available to be dispatched when a new PGx drug is added.

### 3.4 Reporting of results

Ideally, genotyping results should be reported in the patients’ EHR. In case EHRs are available but are not compliant with genetic data, the IT team should upgrade the system to have the necessary features to receive such data. Yet, paper reports may also be considered (Cicali et al., 2022). Another important consideration is the language used to report genotyping data. For example, a report for CYP2C19 may state the result, such as that the genotype is CYP2C19*2, or may describe all evaluated genetic variants. The reporting discrepancies may lead to confusion in interpreting results from different laboratories. With the aim of standardising terms for allele functional status and inferred phenotype for the CPIC guidelines, Caudle et al. (2017) surveyed experts with diverse involvement in at least one area of PGx, and agreed on the following consensus terms: increased, normal, decreased, no, unknown, and uncertain function for allele functional status of all genes; ultrarapid, rapid, normal, intermediate, and poor metabolizer for drug-metabolizing enzymes; increased, normal, decreased, and poor function for the phenotype of transporters; and positive or negative for high-risk genotype status such as for HLA-B. It is hence advisable to include an interpretation of the phenotype associated with the resultant genotype, coupled with a recommendation on what to do next with the drug to be prescribed using language from CPIC or other guidelines. In addition, and if possible, CDS systems should be incorporated to guide the HCP into an informed decision based on genetic data (Caraballo et al., 2017; O'Donnell et al., 2014).

CDS systems can be passive or active. The first generates alerts that are stored in the patient’s EHR for use when needed, while the latter spontaneously delivers alerts pre- and/or post-PGx testing (Hicks et al., 2016; Haidar et al., 2022a). Passive CDS systems require the HCP to remember at the point of care to look for the potential drug-gene interaction, and find the PGx test results in the EHR if available (Haidar et al., 2022a). For active CDS systems, the pre- PGx test alert is triggered when an HCP prescribes an actionable drug at the point of care, and the patient lacks previous genetic test results (Bell et al., 2014). ​ The alert is either displayed directly on the computer screen or by email to direct the HCP to order a genotyping test (Bell et al., 2014). The post- PGx test alert is triggered when a HCP prescribes an actionable drug that coincides with already available high-risk gene data (Bell et al., 2014; Haidar et al., 2022a). ​The alert provides phenotype interpretation, possible drug-gene interaction, if any, and recommended actions, such as changing the drug or dose, or monitoring (Bell et al., 2014; Haidar et al., 2022a).

## 4 Recommendations for the further enhancement of pgx implementation in clinical practice

Some strategies should be put in place to frequently evaluate emerging evidence, continuously audit and evaluate the progress and performance of the program, and integrate research for ethical, legal and social issues (Pirmohamed, 2023). In addition, there is a need to educate HCPs and patients, and to push for more tests’ reimbursement as detailed below.

### 4.1 Education

HCPs and patients are on the receiving end of the clinical PGx implementation process. HCPs, be they physicians or pharmacists, are responsible for the return of results to patients. HCPs and patients are both accountable for reporting back on the outcome of the PGx-guided prescription. They need education to enhance their use and understanding of the whole practice.

#### 4.1.1 Healthcare providers

Survey data have consistently shown that HCPs, despite being generally aware of the importance of PGx and having a positive attitude towards PGx’s ability to improve drug therapy and reduce side effects, few have ordered or recommended PGx testing (Stanek et al., 2012; Abou Diwan et al., 2019; Algahtani, 2020). This lag in PGx clinical implementation is primarily related to the lack of formal education about PGx testing in medical school and postgraduate studies (Stanek et al., 2012; Algahtani, 2020). For instance, a survey among HCPs on PGx education in Europe showed that 83.3% of the participants still lack PGx expertise (Just et al., 2017). Another global survey study was recently done to evaluate the current status of PGx education in medical and pharmacy study programs (Karas Kuzelicki et al., 2019). Results showed that 13.4% had no PGx education among the recruited participants, 19.6% took PGx as an independent elective, and only 10.3% had PGx as a mandatory subject. These results were congruent with survey data collected over a decade ago (2005) and led to the recommendation that PGx should be taught as an integral part of pharmacology curricula (Gurwitz et al., 2005; Karas Kuzelicki et al., 2019). These recommendations are crucial, knowing that education and training increase physicians’ confidence to request PGx tests or use such test results if already available before prescribing drugs (Luzum and Luzum, 2016). ​

Undergraduate education that considers PGx as foundational content can hence contribute to the education of HCP graduates for the integration of PGx into clinical care. Also, training during fellowships or residencies, graduate and postgraduate programs, or certificates can address the PGx knowledge gap. As such, accreditation standards have been developed by the American Society of Health-System Pharmacists for postgraduate year two residence pharmacists to include clinical PGx training (Haidar et al., 2022b).

Besides, continuous education activities are essential to staying up to date. These include just in-time education such as active CDS systems, and PGx programs that provide on-site services, dedicated webpages, and messages to the clinician’s inbox (Freimuth et al., 2017; Williams, 2019). Also, one can build on the already available educational resources, such as those by CPIC. In addition, the National Human Genome Research Institute has supported an Inter-Society Coordinating Committee to develop educational resources aiming to improve the PGx education of HCPs (National Human Genome Research Institute, 2021; Haidar et al., 2022a).

#### 4.1.2 Patients

Patients’ awareness and education are essential drivers for the success of PGx implementation. Available studies suggest that patients have a positive attitude toward PGx implementation and believe in its ability to predict the correct dose, medication efficacy, mild or serious side effects, and explain the family history of medication toxicity (Nielsen and Moldrup, 2007). The general public can also understand specific genetic terminology, yet people cannot comprehend underlying concepts and how this may affect their health (Lea et al., 2011; Haga et al., 2014).

To address this gap, PGx education may be provided through technological tools such as interactive webpages, educational videos, and telehealth sessions (Hoffman et al., 2014; Dunnenberger et al., 2015). Moreover, written reports should be user-friendly. They can include summaries of genetic results in a tabular or graphical format such as infographics (e.g., human icon drawn as running motion representing rapid metabolizer) and icon arrays (e.g., shaded figures reflecting the proportion of affected out of total) (Galesic et al., 2009; Sinayev et al., 2015). Also, simple drawings of pie charts, risk labels, and tri-color-coding systems for risk assessment (red, yellow/orange, and green for high, moderate, and average risk, respectively) (Haga, 2017) are examples of patient-friendly formats. These methods can generate more confidence in patients toward PGx implementation. Finally, adequate counseling should be provided through written reports or in-person contact by well-trained HCPs or genetic counselors if available (Haga, 2017; Haidar et al., 2022a).

### 4.2 Reimbursement considerations

The cost of PGx testing varies between companies and platforms, with the cost of single-gene tests ranging from 100$ to 500$, while the price of a multigene panel test may reach double that of the single-gene test (Anderson et al., 2020; Haidar et al., 2022a). Although single-gene test costs less, patients may require the prescription of several actionable drugs, whereby in this case, multigene panel testing becomes more cost-effective. Nevertheless, most payers are still reluctant to reimburse multigene panel tests due to the lack of evidence of clinical utility for preemptive panel testing (Keeling et al., 2019). Even though the clinical utility of reactive testing using single-gene tests is easier to obtain compared to preemptive multigene panel testing, its reimbursement is still a barrier (Haidar et al., 2022a).

However, reimbursement of PGx in the US may be forthcoming (Empey et al., 2021). Lately, local coverage determinations for the Molecular Diagnostic Services program were established based on earlier decisions made by payers (public and private). They expanded the coverage for some Medicare Administrative Contractors that cover molecular diagnostic tests (US Centers for Medicare and Medicaid Services, 2020). Accordingly, for Medicare patients, local coverage determinations are indicated for PGx tests related to medications that are medically necessary, appropriate, and approved for the patient’s condition and have clinically actionable drug-gene interaction defined by the FDA and CPIC (US Centers for Medicare and Medicaid Services, 2020). Moreover, payer reimbursement policies are evolving, and the availability of specific criteria for PGx testing may increase the probability of coverage (Keeling et al., 2019). For example, the American Medical Association has created several Current Procedure Terminology codes for single-gene PGx tests to detect specific gene variants that impact drug therapy. These codes result in more specific documentation that may increase the chance of PGx test coverage (Hefti and Blanco, 2016). Also, the establishment and demonstration of evidence that PGx improves clinical outcomes, and finding value for PGx testing, as reflected by helping HCPs to decide on therapy for a specific population, may increase the possibility of PGx test reimbursement (Weitzel et al., 2019).

In Europe, the reimbursement systems differ among individual countries (Payne and Annemans, 2013). For the sake of illustration in the Netherlands, all citizens should have a basic healthcare insurance that includes coverage for PGx tests that are ordered to explore causes of ADRs. Moreover, optional reimbursement packages for PGx screening are provided by some healthcare insurers (van der Wouden et al., 2020). In addition, The “G-standaard” Dutch drug database offers information, guidance and standards that are used by different parties in healthcare including health insurers to enhance the infrastructure for national testing programs (Thornley et al., 2021). Finally in England, the National Health Services Genomics Medicine Service that aims to provide genetic services equally among patients is leading innovative projects to allow integration of genetic testing into routine healthcare through supporting research, adequate planning, and reimbursement (Robinson, 2022). Interestingly, a system for reimbursement of PGx testing was suggested whereby it is proposed for medical authorities to develop a “positive medical device” list to control the cost in the market, and impose reimbursement by insurance companies (Vozikis et al., 2016).

Besides coverage by payers, some commercial laboratories provide reimbursement on their panel-based testing by income-based sliding scale payment method, patient assistance programs, or help the patient navigate the reimbursement process. Also, the PGx test can be initially covered or supplemented by institutional support or research funding (Luzum et al., 2017; Cicali et al., 2022).

## 5 Conclusion

This article proposed an approach to designing and implementing clinical PGx in the hospital setting. After test authorization or requirements for testing by the government or drug regulators, putting the plan into action involves several stakeholders, with the hospital leadership supporting the process and communicating with payers, the P&T committee leading the process in collaboration with the hospital laboratory and IT department, and HCPs ordering the test, understanding the results, making the appropriate therapeutic decisions, and explaining them to the patient. We concluded by recommending strategies to further advance the implementation of PGx in practice, such as the need to educate HCPs and patients and to push for more tests’ reimbursement. The reader can refer to Table 4 and learn from the experience of other institutions that have been implementing PGx for years for clinical and or research purposes and adapt some of their approaches concerning the choice of drug-gene pairs, genotyping strategies and methods, integration of results and reports, and educational practices. Several barriers and schemes should be considered before implementing clinical PGx on a big scale (Swift, 2022; Tuteja et al., 2022; Pirmohamed, 2023).

**TABLE 3:** Proposed non-exhaustive list and description of genetic allele variants for the clinical implementation of pharmacogenomics (PGx).

Gene	Allele	rsID	Variation type	Phenotype	MAF from 1,000 genomes with few exceptions^a^ ^,^ ^b^		
					African	Asian	Europe
**ABCG2**	c.421	rs2231142	SNV	Decreased function	0.0129	0.2907	0.0944
**CYP2B6**	*9	rs3745274	SNV	Decreased function	0.3744	0.2153	0.2356
	*18	rs28399499	SNV	No function	0.0825	0.0000	0.0000
	*26	rs3826711	SNV	Decreased function	0.0000	0.0050	0.0000
**CYP2C19**	*2	rs12769205	SNV	No function	0.1967	0.3125	0.1451
		rs4244285	SNV		0.1702	0.3125	0.1451
		rs58973490	SNV		0.0008	0.0000	0.0040
	*3	rs4986893	SNV	No function	0.0023	0.0556	0.000
	*4	rs12248560	SNV	No function	0.2352	0.0149	0.2237
		rs28399504	SNV		0.0000	0.0010	0.0010
	*8	rs41291556	SNV	No function	0.0008	0.0000	0.0030
	*9	rs17884712	SNV	Decreased function	0.0098	0.0000	0.000
	*10	rs6413438	SNV	Decreased function	0.0015	0.0000	0.0000
	*17	rs12248560	SNV	Increased function	0.2352	0.0149	0.2237
**CYP2C9**	*2	rs1799853	SNV	Decreased function	0.0083	0.0010	0.1243
	*3	rs1057910	SNV	No function	0.0023	0.0337	0.0726
	*5	rs28371686	SNV	Decreased function	0.0166	0.0000	0.0000
	*6	rs9332131	Indel	No function	0.0083	0.0000	0.0000
	*8	rs7900194	SNV	Decreased function	0.0530	0.0000	0.0020
	*11	rs28371685	SNV	Decreased function	0.0242	0.0000	0.0020
	*13	rs72558187	SNV	No function	0.0000	0.0030	0.0000
	*14	rs72558189	SNV	Decreased function	0.0000	0.0010	0.0000
	*16	rs72558192	SNV	Decreased function	0.0000	0.0010	0.0000
	*29	rs182132442	SNV	Decreased function	0.0000	0.0030	0.0010
	*31	rs57505750	SNV	Decreased function	0.0015	0.0000	0.0000
	*33	rs200183364	SNV	No function	0.0000	0.0010	0.0000
	*45	rs199523631	SNV	No function	0.0000	0.0000	0.0010
**CYP2D6**	*3	rs35742686	Indel	No function	0.0040	0.0000	0.0189
	*4	rs3892097	SNV	No function	0.0605	0.0020	0.1859
		rs28371703	SNV		0.0204	0.0010	0.1730
		rs28371704	SNV		0.0204	0.0010	0.1730
		rs1058172	SNV		0.0000^a^	0.0211^a^	0.1125^a^
	*5	PV00430	Whole gene deletion	No function	-	-	-
	*6	rs5030655	Indel	No function	0.0008	0.0000	0.0199
	*9	rs5030656	Indel	Decreased function	0.0008	0.0000	0.0258
	*10	rs1065852	SNV	Decreased function	0.1127	0.5714	0.2018
		rs1058164	SNV		0.6344^a^	0.7230^a^	0.5678^a^
		rs1135840	SNV		0.6205^a^	0.7180^a^	0.5673^a^
	*14	rs5030865	SNV	Decreased function	0.0000	0.0099	0.0000
	*17	rs28371706	SNV	Decreased function	0.2179	0.0000	0.0020
		rs1058164	SNV		0.6344^a^	0.7230^a^	0.5678^a^
		rs16947	SNV		0.3398^a^	0.0300^a^	0.3181^a^
		rs1135840	SNV		0.6205^a^	0.7180^a^	0.5673^a^
	*21	rs1058164	SNV	No function	0.6344^a^	0.7230^a^	0.5678^a^
		rs16947	SNV		0.3398^a^	0.0300^a^	0.3181^a^
		rs1135840	SNV		0.6205^a^	0.7180^a^	0.5673^a^
	*29	rs61736512	SNV	Decreased function	0.1097	0.0000	0.0000
		rs59421388	SNV		0.1074	0.0000	0.0000
	*36	rs1065852	SNV	No function	0.1127	0.5714	0.2018
		rs1135822	SNV		0.0003^a^	0.0180^a^	0.0002^a^
		rs1135823	SNV		0.0000	0.0000	0.0010
	*40	rs72549356	Indel	No function	0.0091	0.0000	0.0000
	*41	rs28371725	SNV	Decreased function	0.0182	0.0377	0.9066
	*xN	-	Copy number	Increased function	-	-	-
**CYP3A5**	*3	rs776746	SNV	No function	0.3035^a^	0.7130^a^	0.9299^a^
	*6	rs10264272	SNV	No function	0.1543	0.0000	0.0030
	*7	rs41303343	Indel	No function	0.1180	0.0000	0.0000
**CYP4F2**	*3	rs2108622	SNV	Decreased function	0.0825	0.2143	0.2903
**DPYD**	*2A	rs3918290	SNV	Decreased function	0.0008	0.0000	0.0050
	*13	rs55886062	SNV	Decreased function	0.0000	0.0000	0.0010
	c.2846	rs67376798	SNV	Decreased function	0.0008	0.0000	0.0070
	c.1129-5923	rs75017182	SNV	Decreased function	0.0008	0.0000	0.0239
**HLA-A**	HLA-A*31:01	-	-	High-risk allele	-	0.0556	0.0104
**HLA-B**	HLA-B*15:02	-	-	High-risk allele	-	0.0667	-
	HLA-B*57:01	-	-	High-risk allele	-	0.0111	0.0729
	HLA-B*58:01	-	-	High-risk allele	0.0611	0.0444	0.0104
**MT-RNR1**	c.1555	rs267606617	SNV	Conformational change	-	0.0015^b^	-
	c.1095	rs267606618	SNV	-	-	0.0019^b^	-
**NUDT15**	*2	rs746071566	Indel	No function	0.0015	0.0476	0.0030
	*3	rs116855232	SNV	No function	0.0008	0.0952	0.0020
**SLCO1B1**	*5	rs4149056	SNV	No function	0.0136	0.1230	0.1610
	*9	rs59502379	SNV	No function	0.0408	0.0000	0.0000
	*14	rs11045819	SNV	Increased function	0.0598	0.0030	0.1441
	*15	rs2306283	SNV	No function	0.8177	0.7619	0.4026
**TPMT**	*2	rs1800462	SNV	No function	0.0008	0.0000	0.0060
	*3A	rs1800460	SNV	No function	0.0030	0.0000	0.0278
		rs1142345	SNV		0.0666	0.0218	0.0288
	*3B	rs1800460	SNV	No function	0.0030	0.0000	0.0278
	*3C	rs1142345	SNV	No function	0.0666	0.0218	0.0288
**UGT1A1**	*6	rs4148323	SNV	Decreased function	0.0008	0.1379	0.0070
	*28	rs3064744	Indel: TA (8)	Decreased function	0.4266	0.1290	0.2922
	*36	rs3064744	Indel: TA (6)	Increased function	0.4266	0.1290	0.2922
	*37	rs3064744	Indel: TA (9)	Decreased function	0.4266	0.1290	0.2922
**VKORC1**	c. −1639	rs9923231	SNV	Decreased function	0.0545	0.8849	0.3877

**MAF**, minor allele frequency; **SNV**, single nucleotide variation; **Indel**, Insertion or Deletion.

^a^ From Alfa Allele Frequency.

^b^ From 14KJPN (Allele frequency panel of 14,129 Japanese individuals including the X chromosome).

**TABLE 4:** List and description of few pharmacogenomic (PGx) clinical implementation programs and initiatives.

Description	Choice of drug-gene pairs	Genotyping strategies and methods	Results, EHR integration and CDS	Education
**PMP: PERSONALIZED MEDICINE PROGRAM at the University of Florida Health since 2012** [1-3] https://precisionmedicine.ufhealth.org/about-us/				
➢ It builds and evaluates PGx information for clinical implementation	➢ Based on CPIC guidelines, genotyping of CYP2C19 for clopidogrel was initially launched, followed by TPMT for thiopurines IFNL3 for PEG-IFNα, CYP2D6 for opioids, CYP2D6 and CYP2C19 for SSRIs, and CYP2C19 for PPIs	➢ Involvement of the Pharmacy and Therapeutics Committee	➢ Hospital regulatory body leads the integration of relevant PGx results into EHR and CDS system	➢ Interactive learning opportunities focusing on review of evidence and development of clinical recommendations
➢ It also identifies and addresses common challenges		➢ Preemptive genotyping	➢ Rapid reporting of results into the EHR (Epic) after 2–3 days	➢ Education of target audience by provider group. Provision of material (printed and online) for clinicians and patients
	➢ Choice was also based on FDA product label, presence of no function genetic variants or common allele frequency, potential to prevent adverse drug events, available evidence supporting genotype-guided dosing recommendations, and physician request	➢ Life technologies Quant Studio Open Array technology. Chip-based genotyping	➢ Use of Best Practice Advisories (BPA) CDS system that provides interpretation and clinical recommendations based on patient’s genetic results	➢ Development of a novel elective course for pharmacy students
				➢ Development of accredited post-graduate training programs in PGx
				➢ Publication of a newsletter titled “SNP.its”
**PG4KDS: PHARMACOGENETICS FOR KIDS at the St. Jude Children’s Research Hospital since 2011** [3-5] https://www.stjude.org/treatment/clinical-trials/pg4kds-pharmaceutical-science.html				
➢ It targets children with cancer	➢ Based on CPIC guidelines, genotyping for CYP2C19, CYP2D6, TPMT, and SLCO1B1 were initially chosen coupled with 12 high-risk drugs. After that, DPYD, UGT1A1, CYP3A5, CYP2C9, NUD15, RYR1, mt-RNR1, CACNA1S, G6PD, and CYP2B6 were genotyped, which resulted in therapeutic guidance for 66 drugs	➢ Creation of a subcommittee of the hospital Pharmacy and Therapeutics Committee for PGx oversight	➢ Test results are first displayed in a specialty flow sheet tab. Some are then moved to the EHR with phenotype description, interpretation, and implication	➢ Development of accredited post-graduate programs in clinical PGx
➢ It preemptively analyzes patients’ DNA for a large number of gene variants, generates reports, and incorporates relevant PGx data in EHR coupled with CDS.	➢ Focus on drugs for children with cancer	➢ Launching of a research protocol with informed consent to implement preemptive genotyping strategy with integration into the EHR.	➢ Consultation notes are available for clinicians with basic PGx knowledge as a passive decision support tool	➢ Website includes presentations and publications on the implemented drug-gene pairs
		➢ Initially started with the Affymetrix DMET Plus assay, later moved to the right patient right drug (RPRD) diagnostic with the PharmacoScan array	➢ Results and consultations are available in the patient’s online portal	
			➢ Active CDS alerts with relevant drug prescriptions	
**PREDICT: PHARMACOGENOMICS RESOURCE FOR ENHANCED DECISIONS IN CARE AND TREATMENT at Vanderbilt University Medical Center since 2010** [3, 5, 6]				
https://www.vumc.org/predict-pdx/				
➢ It chooses drug-gene pairs, genotypes, filters, interprets, and incorporates PGx data and CDS in EHRs to be accessible for healthcare providers in routine care	➢ Based on CPIC guidelines, CYP2C19 was initially genotyped for clopidogrel followed by CYP2C9 and VKORC1 for warfarin therapy	➢ Involvement of the Pharmacy and Therapeutics Committee	➢ Results are entered by laboratory staff to the laboratory information system (Cerner Millennium Helix^®^ module)	➢ Development of “My Drug Genome” website
	➢ Focus on drugs for cardiovascular diseases	➢ Preemptive genotyping	➢ Results of discrete variants are found on the EHR (Epic) as patient friendly version through My Health At Vanderbilt (MHAV)	➢ Support for the development of a Massive Open Online Course (MOOC) on PGx
		➢ TaqMan^®^ chemistry-based platforms such as Oper QuantStudio™ 12K Flex Real-Time OpenArray Polymerase Chain Reaction (PCR) platform for more than 50 samples/day	➢ Application of end-to-end CDS system to help in interpretation of results and guidance in medication/dose selection	➢ Offering of a post-doctoral fellowship program and training in pharmacogenomics
			➢ Use of drug-gene interaction knowledge to interpret genotype-phenotype relation and linking of a specific CDS to a specific genetic result	
**RIGHT: RIGHT DRUG, RIGHT DOSE, RIGHT TIME at the Mayo Clinic since 2013** [3, 5, 7]				
https://www.mayo.edu/research/centers-programs/center-individualized-medicine/research/clinical-studies/right-10k				
➢ It evaluates available PGx studies and guidelines	➢ Based on CPIC guidelines, genotyping of SLCO1B1 for simvastatin was initially done followed by CYP2C19 for clopidogrel, IFNL2 for interferon, CYP2D6 for tramadol, tamoxifen and codeine, HLA-B*1,502 for carbamazepine and abacavir, and TPMT for thiopurines	➢ Involvement of the pharmaceutical formulary committee in approving drug-gene pairs and incorporation of results with CDS.	➢ Storage of molecular diagnostic laboratory results in EHR.	➢ The CDS rules provide information on drug-gene pair at point of care as a “Just in Time” support system
➢ It genotypes and incorporates PGx data and CDS into EHR to be accessible for healthcare providers	➢ Choice was also based on commonly prescribed drugs containing actionable PGx variants, FDA list of PGx biomarkers, PharmGKB list of genes and drugs, Indiana University Drug Interactions website, articles published on the subject of PGx, and current PGx tests offered by the Mayo Clinic’s Department of Laboratory Medicine and Pathology	➢ PGx implementation model following this sequence: Institutional leadership support, Pharmacogenomics governance, Clinical approval, Laboratory results, Pharmacogenomics education, Pharmacogenomics knowledge, CDS-EHR implementation, and long-term maintenance	➢ Development and maintenance of CDS rules that involve conversion of variants to standard notation and interpretation, workflow analysis, and data mapping	➢ Information and interpretation of PGx testing is available for patients through “Online Patient Services account”
		➢ Development of Mayo Clinic Biobank Community Advisory Board (CAB) for recruitment and consenting patients	➢ CDS rules are implemented for interpreting results, prescribing decisions, and providing actionable alert messages	➢ Development of “Ask Mayo Expert” for patient education
		➢ Preemptive research genotyping through the RIGHT protocol	➢ Active CDS alerts are developed in the computerized physician order entry (CPOE) applications	➢ Establishment of grand rounds, presentations, online modules, videos, brochures, and links to results through the patient’s portal
		➢ Use of PGRN-Seq technique		➢ Offering of a post-doctoral fellowship program and training in PGx
**The 1200 PATIENT PROJECT at the University of Chicago Center for Personalized Therapeutics since 2011** [3, 5, 8]https://cpt.uchicago.edu/1200-patients-project/				
➢ It assesses the effectiveness and feasibility of applying preemptive PGx testing in clinical settings	➢ Large number of germline polymorphisms for outpatient medical care	➢ Research study targeting 1,200 patients for the implementation of preemptive genotyping with informed consent	➢ Results, interpretation and education are available through research web-portal or genomic prescribing system (GPS)	➢ Development of “YourPGx Portal”
➢ It also evaluates the impact of using PGx results on prescription decisions and patients’ outcome	➢ Participants should be taking 1 to 6 prescription medications of interest	➢ Use of ‘ADME pharmacogenomics panel’ and custom Sequenom panel		➢ Offering of a post-doctoral fellowship program and training in PGx
	➢ Choice of genes based on published clinical evidence for their PGx role			
**U-PGx: UBIQUITOUS PHARMACOGENOMICS Consortium in Europe since 2016** (The Netherland, United Kingdom, Germany, Sweden, Austria, France, Italy, Spain, Greece, Slovenia) [3] https://uPGx.eu/				
➢ It evaluates the cost-effectiveness and impact of preemptive PGx implementation in Europe on patient outcome by conducting ‘The Preemptive Pharmacogenomic testing for Prevention of adverse drug reactions (PREPARE)’ study	➢ Panel of 50 variants in 13 pharmacogenes that have actionable drug-gene interaction based on the Dutch Pharmacogenetics Working Group (DPWG) guidelines	➢ Research study (PREPARE) on the preemptive implementation of a panel of pharmacogenes covering several therapeutic areas	➢ Medication Safety Code system: “Safety-Code card” and Genetic Information Management Suite for physicians	➢ Established E-learning PGx programs for healthcare providers
	➢ The 13 genes evaluated are: CYP1A2, CYP2B6, CYP2C9, CYP2C19, CYP2D6, CYP3A5, DPYD, F5, HLA‐ B*5701, SLCO1B1, TPMT, UGT1A1, VKORC1	➢ Use of SNPline platform		
	➢ For patients being started on a drug of interest that has clinically relevant genetic interaction with the genes mentioned. The chosen drugs are very similar to the ones listed in Table 2			

EHR, electronic health records; CDS, clinical decision support.

1. Johnson, J.A., et al., Institutional profile: University of Florida and Shands Hospital Personalized Medicine Program: clinical implementation of pharmacogenetics. Pharmacogenomics, 2013. **14** (7): p. 723-6.

2. Cavallari, L.H., et al., Institutional profile: University of Florida Health Personalized Medicine Program. pharmacogenomics, 2017. **18** (5): p. 421-426.

3. Van der Wouden, C.H., et al., Implementing Pharmacogenomics in Europe: Design and Implementation Strategy of the Ubiquitous Pharmacogenomics Consortium. clin pharmacol ther, 2017. **101** (3): p. 341-358.

4. Haidar, C.E., et al., Advancing Pharmacogenomics from Single-Gene to Preemptive Testing. annu rev genomics hum genet, 2022. **23**: p. 449-473.

5. Luzum, J.A., et al., The Pharmacogenomics Research Network Translational Pharmacogenetics Program: Outcomes and Metrics of Pharmacogenetic Implementations Across Diverse Healthcare Systems. clin pharmacol ther, 2017. 102 (3): p. 502-510.

6. Liu, M., et al., A Tutorial for Pharmacogenomics Implementation Through End-to-End Clinical Decision Support Based on Ten Years of Experience from PREDICT., clin pharmacol ther, 2021. 109 (1): p. 101-115.

7. Bielinski, S.J., et al., Preemptive genotyping for personalized medicine: design of the right drug, right dose, right time-using genomic data to individualize treatment protocol. Mayo Clin Proc, 2014. 89 (1): p. 25-33.

8. O'Donnell, P.H., et al., The 1,200 patients project: creating a new medical model system for clinical implementation of pharmacogenomics. Clin Pharmacol Ther, 2012. 92 (4): p. 446-9.

## References

[B1] Abou Diwan E. Zeitoun R. .I. Abou Haidar L. Cascorbi I. Khoueiry Zgheib N. (2019). Implementation and obstacles of pharmacogenetics in clinical practice: An international survey. Br. J. Clin. Pharmacol. 85 (9), 2076–2088. 10.1111/bcp.13999 31141189 PMC6710530

[B2] Algahtani M. (2020). Knowledge, perception, and application of pharmacogenomics among hospital pharmacists in Saudi arabia. Risk Manag. Healthc. Policy 13, 1279–1291. 10.2147/RMHP.S267492 32904476 PMC7455604

[B3] Anderson H. .D. Crooks K. .R. Kao D. .P. Aquilante C. .L. (2020). The landscape of pharmacogenetic testing in a US managed care population. Genet. Med. 22 (7), 1247–1253. 10.1038/s41436-020-0788-3 32291400 PMC7332417

[B4] Association of Molecular Pathology (AMP) (1995). Association of molecular Pathology (AMP). Available at: https://www.amp.org/.

[B5] Bartlett G. Antoun J. Zgheib N. .K. (2012). Theranostics in primary care: Pharmacogenomics tests and beyond. Expert Rev. Mol. Diagn 12 (8), 841–855. 10.1586/erm.12.115 23249202

[B6] Bell G. .C. Crews K. .R. Wilkinson M. .R. Haidar C. .E. Hicks J. .K. Baker D. .K. (2014). Development and use of active clinical decision support for preemptive pharmacogenomics. J. Am. Med. Inf. Assoc. 21 (1), e93–e99. 10.1136/amiajnl-2013-001993 PMC3957400 23978487

[B7] Braten L. .S. Haslemo T. Jukic M. .M. Ivanov M. Ingelman-Sundberg M. Molden E. (2021). A novel CYP2C-haplotype associated with ultrarapid metabolism of escitalopram. Clin. Pharmacol. Ther. 110 (3), 786–793. 10.1002/cpt.2233 33759177

[B8] Braten L. .S. Ingelman-Sundberg M. Jukic M. .M. Molden E. Kringen M. .K. (2022). Impact of the novel CYP2C:TG haplotype and CYP2B6 variants on sertraline exposure in a large patient population. Clin. Transl. Sci. 15 (9), 2135–2145. 10.1111/cts.13347 35668575 PMC9468554

[B9] Bristol L. .A. (2002). A regulatory protocol for pharmacogenomics services. Pharmacogenomics J. 2 (2), 83–86. 10.1038/sj.tpj.6500079 12049179

[B10] Brooks G. .A. Tapp S. Daly A. .T. Busam J. .A. Tosteson A. .N. .A. (2022). Cost-effectiveness of DPYD genotyping prior to fluoropyrimidine-based adjuvant chemotherapy for colon cancer. Clin. Colorectal Cancer 21 (3), e189–e195. 10.1016/j.clcc.2022.05.001 35668003 PMC10496767

[B11] Canadian Pharmacogenomics Network for Drug Safety (CPNDS) (2004). Canadian pharmacogenomics network for drug safety (CPNDS). Available at: https://cpnds.ubc.ca/.

[B12] Caraballo P. .J. Hodge L. .S. Bielinski S. .J. Stewart A. .K. Farrugia G. Schultz C. .G. (2017). Multidisciplinary model to implement pharmacogenomics at the point of care. Genet. Med. 19 (4), 421–429. 10.1038/gim.2016.120 27657685 PMC5362352

[B13] Caudle K. .E. Dunnenberger H. .M. Freimuth R. .R. Peterson J. .F. Burlison J. .D. Whirl-Carrillo M. (2017). Standardizing terms for clinical pharmacogenetic test results: Consensus terms from the clinical pharmacogenetics implementation Consortium (CPIC). Genet. Med. 19 (2), 215–223. 10.1038/gim.2016.87 27441996 PMC5253119

[B14] Caudle K. .E. Gammal R. .S. Whirl-Carrillo M. Hoffman J. .M. Relling M. .V. Klein T. .E. (2016). Evidence and resources to implement pharmacogenetic knowledge for precision medicine. Am. J. Health Syst. Pharm. 73 (23), 1977–1985. 10.2146/ajhp150977 27864205 PMC5117674

[B15] Centers for Disease Control and Prevention (2000). ACCE model process for evaluating genetic tests. Available at: https://www.cdc.gov/genomics/gtesting/acce/index.htm.

[B16] Chanfreau-Coffinier C. Hull L. .E. Lynch J. .A. DuVall S. .L. Damrauer S. .M. Cunningham F. .E. (2019). Projected prevalence of actionable pharmacogenetic variants and level A drugs prescribed among US veterans health administration pharmacy users. JAMA Netw. Open 2 (6), e195345. 10.1001/jamanetworkopen.2019.5345 31173123 PMC6563578

[B17] Cicali E. .J. Lemke L. Al Alshaykh H. Nguyen K. Cavallari L. .H. Wiisanen K. (2022). How to implement a pharmacogenetics service at your institution. J. Am. Coll. Clin. Pharm. 5 (11), 1161–1175. 10.1002/jac5.1699 36589694 PMC9799247

[B18] Clinical pharmacogenetics implemetation Consortium (CPIC).(2009) Clinical pharmacogenetics implemetation Consortium (CPIC) Available at: https://cpicpgx.org/ .

[B19] Dandara C. Masimirembwa C. Haffani Y. .Z. Ogutu B. Mabuka J. Aklillu E. (2019). African Pharmacogenomics Consortium: Consolidating pharmacogenomics knowledge, capacity development and translation in Africa: Consolidating pharmacogenomics knowledge, capacity development and translation in Africa. AAS Open Res. 2, 19. 10.12688/aasopenres.12965.1 32382701 PMC7194139

[B20] Dean A. .S. Zignol M. Cabibbe A. .M. Falzon D. Glaziou P. Cirillo D. .M. (2020). Prevalence and genetic profiles of isoniazid resistance in tuberculosis patients: A multicountry analysis of cross-sectional data. PLoS Med. 17 (1), e1003008. 10.1371/journal.pmed.1003008 31961877 PMC6974034

[B21] Deenen M. .J. Meulendijks D. Cats A. Sechterberger M. .K. Severens J. .L. Boot H. (2016). Upfront genotyping of DPYD*2A to individualize fluoropyrimidine therapy: A safety and cost analysis. J. Clin. Oncol. 34 (3), 227–234. 10.1200/JCO.2015.63.1325 26573078

[B22] Dunnenberger H. .M. Crews K. .R. Hoffman J. .M. Caudle K. .E. Broeckel U. Howard S. .C. (2015). Preemptive clinical pharmacogenetics implementation: Current programs in five US medical centers. Annu. Rev. Pharmacol. Toxicol. 55, 89–106. 10.1146/annurev-pharmtox-010814-124835 25292429 PMC4607278

[B23] Ehmann F. Caneva L. Papaluca M. (2014). European Medicines Agency initiatives and perspectives on pharmacogenomics. Br. J. Clin. Pharmacol. 77 (4), 612–617. 10.1111/bcp.12319 24433361 PMC3971978

[B24] El Shamieh S. Zgheib N. .K. (2022). Pharmacogenetics in developing countries and low resource environments. Hum. Genet. 141 (6), 1159–1164. 10.1007/s00439-021-02260-9 33564904

[B25] Electronic Medical Records and Genomics Network (e-MERGE) (2007). Electronic medical records and genomics (eMERGE) network. Available at: https://www.genome.gov/Funded-Programs-Projects/Electronic-Medical-Records-and-Genomics-Network-eMERGE.

[B26] Empey P. .E. Pratt V. .M. Hoffman J. .M. Caudle K. .E. Klein T. .E. (2021). Expanding evidence leads to new pharmacogenomics payer coverage. Genet. Med. 23 (5), 830–832. 10.1038/s41436-021-01117-w 33627827 PMC8222707

[B27] Endrullat C. Glokler J. Franke P. Frohme M. (2016). Standardization and quality management in next-generation sequencing. Appl. Transl. Genom 10, 2–9. 10.1016/j.atg.2016.06.001 27668169 PMC5025460

[B28] European co-operation for Accreditation (2022). European co-operation for Accreditation. Available at: https://european-accreditation.org/.

[B29] European Medicinal Agency (Ema) (1995). [Available from: https://www.ema.europa.eu/en.

[B30] European Medicines Agency (2010). Co-development of pharmacogenomic biomarkers and assays in the context of drug development - scientific guideline. Available at: https://www.ema.europa.eu/en/co-development-pharmacogenomic-biomarkers-assays-context-drug-development-scientific-guideline.

[B31] European Medicines Agency (1998). Plavix. Available at: https://www.ema.europa.eu/en/medicines/human/EPAR/plavix.

[B32] Freimuth R. .R. Formea C. .M. Hoffman J. .M. Matey E. Peterson J. .F. Boyce R. .D. (2017). Implementing genomic clinical decision support for drug-based precision medicine. CPT Pharmacometrics Syst. Pharmacol. 6 (3), 153–155. 10.1002/psp4.12173 28109071 PMC5351408

[B33] Gaedigk A. Casey S. .T. Whirl-Carrillo M. Miller N. .A. Klein T. .E. (2021). Pharmacogene variation Consortium: A global resource and repository for pharmacogene variation. Clin. Pharmacol. Ther. 110 (3), 542–545. 10.1002/cpt.2321 34091888 PMC8725060

[B34] Gaedigk A. Ingelman-Sundberg M. Miller N. .A. Leeder J. .S. Whirl-Carrillo M. Klein T. .E. (2018). The pharmacogene variation (PharmVar) Consortium: Incorporation of the human cytochrome P450 (CYP) allele nomenclature database. Clin. Pharmacol. Ther. 103 (3), 399–401. 10.1002/cpt.910 29134625 PMC5836850

[B35] Gaedigk A. Whirl-Carrillo M. Pratt V. .M. Miller N. .A. Klein T. .E. (2020). PharmVar and the landscape of pharmacogenetic resources. Clin. Pharmacol. Ther. 107 (1), 43–46. 10.1002/cpt.1654 31758698 PMC6925620

[B36] Galesic M. Garcia-Retamero R. Gigerenzer G. (2009). Using icon arrays to communicate medical risks: Overcoming low numeracy. Health Psychol. 28 (2), 210–216. 10.1037/a0014474 19290713

[B37] Grant A. .D. De Cock K. .M. (1998). The growing challenge of HIV/AIDS in developing countries. Br. Med. Bull. 54 (2), 369–381. 10.1093/oxfordjournals.bmb.a011694 9830203

[B38] Greenwood B. (2004). Treating malaria in Africa. BMJ 328 (7439), 534–535. 10.1136/bmj.328.7439.534 15001479 PMC381032

[B39] Gurwitz D. Lunshof J. .E. Dedoussis G. Flordellis C. .S. Fuhr U. Kirchheiner J. (2005). Pharmacogenomics education: International Society of Pharmacogenomics recommendations for medical, pharmaceutical, and health schools deans of education. Pharmacogenomics J. 5 (4), 221–225. 10.1038/sj.tpj.6500312 15852053

[B40] Haga S. .B. (2017). Educating patients and providers through comprehensive pharmacogenetic test reports. Pharmacogenomics 18 (11), 1047–1050. 10.2217/pgs-2017-0088 28686141 PMC5591460

[B41] Haga S. .B. Mills R. Bosworth H. (2014). Striking a balance in communicating pharmacogenetic test results: Promoting comprehension and minimizing adverse psychological and behavioral response. Patient Educ. Couns. 97 (1), 10–15. 10.1016/j.pec.2014.06.007 24985359 PMC4162835

[B42] Haidar C. .E. Crews K. .R. Hoffman J. .M. Relling M. .V. Caudle K. .E. (2022). Advancing pharmacogenomics from single-gene to preemptive testing. Annu. Rev. Genomics Hum. Genet. 23, 449–473. 10.1146/annurev-genom-111621-102737 35537468 PMC9483991

[B43] Haidar C. .E. Petry N. Oxencis C. Douglas J. .S. Hoffman J. .M. (2022). ASHP statement on the pharmacist's role in clinical pharmacogenomics. Am. J. Health Syst. Pharm. 79 (8), 704–707. 10.1093/ajhp/zxab339 34487145

[B44] Hartzler A. McCarty C. .A. Rasmussen L. .V. Williams M. .S. Brilliant M. Bowton E. .A. (2013). Stakeholder engagement: A key component of integrating genomic information into electronic health records. Genet. Med. 15 (10), 792–801. 10.1038/gim.2013.127 24030437 PMC3909653

[B45] Hefti E. Blanco J. .G. (2016). Documenting pharmacogenomic testing with CPT codes. J. AHIMA 87 (1), 56–59. PMC4998735 27055343

[B46] Hicks J. .K. Dunnenberger H. .M. Gumpper K. .F. Haidar C. .E. Hoffman J. .M. (2016). Integrating pharmacogenomics into electronic health records with clinical decision support. Am. J. Health Syst. Pharm. 73 (23), 1967–1976. 10.2146/ajhp160030 27864204 PMC5117634

[B47] Hicks J. .K. El Rouby N. Ong H. .H. Schildcrout J. .S. Ramsey L. .B. Shi Y. (2021). Opportunity for genotype-guided prescribing among adult patients in 11 US health systems. Clin. Pharmacol. Ther. 110 (1), 179–188. 10.1002/cpt.2161 33428770 PMC8217370

[B48] Hoffman J. .M. Haidar C. .E. Wilkinson M. .R. Crews K. .R. Baker D. .K. Kornegay N. .M. (2014). PG4KDS: A model for the clinical implementation of pre-emptive pharmacogenetics. Am. J. Med. Genet. C Semin. Med. Genet. 166C (1), 45–55. 10.1002/ajmg.c.31391 24619595 PMC4056586

[B49] Implementing Genomics in Practice (IGNITE) (2013). Implementing genomics in practice (IGNITE). Available at: https://www.genome.gov/Funded-Programs-Projects/Implementing-Genomics-in-Practice-IGNITE.

[B50] International Laboratory Accreditation Cooperation (2022). International laboratory accreditation cooperation. Available at: https://ilac.org/.

[B51] Johnson J. .A. Caudle K. .E. Gong L. Whirl-Carrillo M. Stein C. .M. Scott S. .A. (2017). Clinical pharmacogenetics implementation Consortium (CPIC) guideline for pharmacogenetics-guided warfarin dosing: 2017 update. Clin. Pharmacol. Ther. 102 (3), 397–404. 10.1002/cpt.668 28198005 PMC5546947

[B52] Just K. .S. Steffens M. Swen J. .J. Patrinos G. .P. Guchelaar H. .J. Stingl J. .C. (2017). Medical education in pharmacogenomics-results from a survey on pharmacogenetic knowledge in healthcare professionals within the European pharmacogenomics clinical implementation project Ubiquitous Pharmacogenomics (U-PGx). Eur. J. Clin. Pharmacol. 73 (10), 1247–1252. 10.1007/s00228-017-2292-5 28669097 PMC5599468

[B53] Karamperis K. Koromina M. Papantoniou P. Skokou M. Kanellakis F. Mitropoulos K. (2021). Economic evaluation in psychiatric pharmacogenomics: A systematic review. Pharmacogenomics J. 21 (4), 533–541. 10.1038/s41397-021-00249-1 34215853

[B54] Karas Kuzelicki N. Prodan Zitnik I. Gurwitz D. Llerena A. Cascorbi I. Siest S. (2019). Pharmacogenomics education in medical and pharmacy schools: Conclusions of a global survey. Pharmacogenomics 20 (9), 643–657. 10.2217/pgs-2019-0009 31250730

[B55] Keeling N. .J. Rosenthal M. .M. West-Strum D. Patel A. .S. Haidar C. .E. Hoffman J. .M. (2019). Preemptive pharmacogenetic testing: Exploring the knowledge and perspectives of US payers. Genet. Med. 21 (5), 1224–1232. 10.1038/gim.2017.181 31048813 PMC5920773

[B56] Kennedy C. O'Reilly K. Medley A. Sweat M. (2007). The impact of HIV treatment on risk behaviour in developing countries: A systematic review. AIDS Care 19 (6), 707–720. 10.1080/09540120701203261 17573590

[B57] Kimpton J. .E. Carey I. .M. Threapleton C. .J. .D. Robinson A. Harris T. Cook D. .G. (2019). Longitudinal exposure of English primary care patients to pharmacogenomic drugs: An analysis to inform design of pre-emptive pharmacogenomic testing. Br. J. Clin. Pharmacol. 85 (12), 2734–2746. 10.1111/bcp.14100 31454087 PMC6955399

[B58] Koutsilieri S. Tzioufa F. Sismanoglou D. .C. Patrinos G. .P. (2020). Unveiling the guidance heterogeneity for genome-informed drug treatment interventions among regulatory bodies and research consortia. Pharmacol. Res. 153, 104590. 10.1016/j.phrs.2019.104590 31830522

[B59] Krebs K. Milani L. (2019). Translating pharmacogenomics into clinical decisions: Do not let the perfect be the enemy of the good. Hum. Genomics 13 (1), 39. 10.1186/s40246-019-0229-z 31455423 PMC6712791

[B60] Lea D. .H. Kaphingst K. .A. Bowen D. Lipkus I. Hadley D. .W. (2011). Communicating genetic and genomic information: Health literacy and numeracy considerations. Public Health Genomics 14 (4-5), 279–289. 10.1159/000294191 20407217 PMC2909377

[B61] Luzum J. .A. Luzum M. .J. (2016). Physicians' attitudes toward pharmacogenetic testing before and after pharmacogenetic education. Per Med. 13 (2), 119–127. 10.2217/pme.15.57 29749904 PMC5907693

[B62] Luzum J. .A. Pakyz R. .E. Elsey A. .R. Haidar C. .E. Peterson J. .F. Whirl-Carrillo M. (2017). The pharmacogenomics research network translational pharmacogenetics program: Outcomes and Metrics of pharmacogenetic implementations across diverse healthcare systems. Clin. Pharmacol. Ther. 102 (3), 502–510. 10.1002/cpt.630 28090649 PMC5511786

[B63] Luzum J. .A. Petry N. Taylor A. .K. Van Driest S. .L. Dunnenberger H. .M. Cavallari L. .H. (2021). Moving pharmacogenetics into practice: It's all about the evidence. Clin. Pharmacol. Ther. 110 (3), 649–661. 10.1002/cpt.2327 34101169 PMC8376790

[B64] Marais B. .J. Lonnroth K. Lawn S. .D. Migliori G. .B. Mwaba P. Glaziou P. (2013). Tuberculosis comorbidity with communicable and non-communicable diseases: Integrating health services and control efforts. Lancet Infect. Dis. 13 (5), 436–448. 10.1016/S1473-3099(13)70015-X 23531392

[B65] Medwid S. Kim R. .B. (2022). Implementation of pharmacogenomics: Where are we now? Br. J. Clin. Pharmacol. 10.1111/bcp.15591 36366858

[B66] Mehta D. Uber R. Ingle T. Li C. Liu Z. Thakkar S. (2020). Study of pharmacogenomic information in FDA-approved drug labeling to facilitate application of precision medicine. Drug Discov. Today 25 (5), 813–820. 10.1016/j.drudis.2020.01.023 32032705

[B67] Mitri Z. Esmerian M. .O. Simaan J. .A. Sabra R. Zgheib N. .K. (2010). Pharmacogenetics and personalized medicine: The future for drug prescribing. J. Med. Liban. 58 (2), 101–104. 20549897

[B68] National Center for Biotechnology Information (NCBI) (1999). dbSNP database. [Available from: https://www.ncbi.nlm.nih.gov/snp/.

[B69] National Center for Biotechnology Information (NCBI) (2012). Genetic testing Registry (GTR). Available at: https://www.ncbi.nlm.nih.gov/gtr/.

[B70] National genomic test directory (2022). National genomic test directory. Available at: https://www.england.nhs.uk/publication/national-genomic-test-directories/.

[B71] National Human Genome Research Institute (2021). Educational resources. Available at: https://www.genome.gov/About-Genomics/Educational-Resources.

[B72] Nicholson W. .T. Formea C. .M. Matey E. .T. Wright J. .A. Giri J. Moyer A. .M. (2021). Considerations when applying pharmacogenomics to your practice. Mayo Clin. Proc. 96 (1), 218–230. 10.1016/j.mayocp.2020.03.011 33308868

[B73] Nielsen L. .F. Moldrup C. (2007). The diffusion of innovation: Factors influencing the uptake of pharmacogenetics. Community Genet. 10 (4), 231–241. 10.1159/000106562 17895629

[B74] O'Donnell P. .H. Danahey K. Jacobs M. Wadhwa N. .R. Yuen S. Bush A. (2014). Adoption of a clinical pharmacogenomics implementation program during outpatient care--initial results of the University of Chicago "1,200 Patients Project. Am. J. Med. Genet. C Semin. Med. Genet. 166C (1), 68–75. 10.1002/ajmg.c.31385 24616296 PMC4000170

[B75] Payne K. Annemans L. (2013). Reflections on market access for personalized medicine: Recommendations for Europe. Value Health 16 (6), S32–S38. 10.1016/j.jval.2013.06.010 24034310

[B76] Penas L. .E. (2023). Clinical use of pre-emptive pharmacogenetic programmes. Lancet 401 (10374), 320–321. 10.1016/S0140-6736(22)02461-8 36739126

[B77] Pharmacogene Variation Consortium (PharmVar) (2000). Pharmacogene variation Consortium (PharmVar). Available at: https://www.pharmvar.org/.

[B78] Pharmacogenomics Clinical Annotation Tool (PharmCat) (2022). Pharmacogenomics clinical annotation tool (PharmCat). Available at: https://pharmcat.org/.

[B79] Pharmacogenomics Knowledge Base (PharmGKB).(2022) Clinical guideline annotations. Available at: https://www.pharmgkb.org/guidelineAnnotations.

[B80] Pharmacogenomics Knowledge Base (PharmGKB) (2017). Drug label annotations. Available at: https://www.pharmgkb.org/labelAnnotations.

[B81] Pharmacogenomics Knowledge Base (PharmGKB) (2001). Pharmacogenomics knowledge base (PharmGKB). Available at: https://www.pharmgkb.org/.

[B82] Pharmacogenomics Knowledge Base (PharmGKB) (2020). VIPs: Very important pharmacogenes. Available at: https://www.pharmgkb.org/vips.

[B83] Pharmacogenomics Research Network (PGRN) (1998). [Available from: https://www.pgrn.org/.

[B84] Picard N. Boyer J. .C. Etienne-Grimaldi M. .C. Barin-Le Guellec C. Thomas F. Loriot M. .A. (2017). Pharmacogenetics-based personalized therapy: Levels of evidence and recommendations from the French Network of Pharmacogenetics (RNPGx). Therapie 72 (2), 185–192. 10.1016/j.therap.2016.09.014 28237406

[B85] Pirmohamed M. (2023). Pharmacogenomics: Current status and future perspectives. Nat. Rev. Genet. 10.1038/s41576-022-00572-8 36707729

[B86] Pratt V. .M. Del Tredici A. .L. Hachad H. Ji Y. Kalman L. .V. Scott S. .A. (2018). Recommendations for clinical CYP2C19 genotyping allele selection: A report of the association for molecular Pathology. J. Mol. Diagn 20 (3), 269–276. 10.1016/j.jmoldx.2018.01.011 29474986

[B87] Rigter T. Jansen M. .E. de Groot J. .M. Janssen S. .W. .J. Rodenburg W. Cornel M. .C. (2020). Implementation of pharmacogenetics in primary care: A multi-stakeholder perspective. Front. Genet. 11, 10. 10.3389/fgene.2020.00010 32076434 PMC7006602

[B88] Robinson J. (2022). Everything you need to know about the NHS genomic medicine service. Available at: https://pharmaceutical-journal.com/article/feature/everything-you-need-to-know-about-the-nhs-genomic-medicine-service.

[B89] Roden D. .M. Van Driest S. .L. Mosley J. .D. Wells Q. .S. Robinson J. .R. Denny J. .C. (2018). Benefit of preemptive pharmacogenetic information on clinical outcome. Clin. Pharmacol. Ther. 103 (5), 787–794. 10.1002/cpt.1035 29377064 PMC6134843

[B90] Rollinson V. Turner R. Pirmohamed M. (2020). Pharmacogenomics for primary care: An overview. Genes. (Basel). 11 (11), 1337. 10.3390/genes11111337 33198260 PMC7696803

[B91] Rubinstein W. .S. Maglott D. .R. Lee J. .M. Kattman B. .L. Malheiro A. .J. Ovetsky M. (2013). The NIH genetic testing registry: A new, centralized database of genetic tests to enable access to comprehensive information and improve transparency. Nucleic Acids Res. 41 (2012), D925–D935. 10.1093/nar/gks1173 23193275 PMC3531155

[B92] Samwald M. Xu H. Blagec K. Empey P. .E. Malone D. .C. Ahmed S. .M. (2016). Incidence of exposure of patients in the United States to multiple drugs for which pharmacogenomic guidelines are available. PLoS One 11 (10), e0164972. 10.1371/journal.pone.0164972 27764192 PMC5072717

[B93] Sangkuhl K. Whirl-Carrillo M. Whaley R. .M. Woon M. Lavertu A. Altman R. .B. (2020). Pharmacogenomics clinical annotation tool (PharmCAT). Clin. Pharmacol. Ther. 107 (1), 203–210. 10.1002/cpt.1568 31306493 PMC6977333

[B94] Schildcrout J. .S. Denny J. .C. Bowton E. Gregg W. Pulley J. .M. Basford M. .A. (2012). Optimizing drug outcomes through pharmacogenetics: A case for preemptive genotyping. Clin. Pharmacol. Ther. 92 (2), 235–242. 10.1038/clpt.2012.66 22739144 PMC3785311

[B95] Scott S. .A. Sangkuhl K. Stein C. .M. Hulot J. .S. Mega J. .L. Roden D. .M. (2013). Clinical pharmacogenetics implementation Consortium guidelines for CYP2C19 genotype and clopidogrel therapy: 2013 update. Clin. Pharmacol. Ther. 94 (3), 317–323. 10.1038/clpt.2013.105 23698643 PMC3748366

[B96] Shekhani R. Steinacher L. Swen J. .J. Ingelman-Sundberg M. (2020). Evaluation of current regulation and guidelines of pharmacogenomic drug labels: Opportunities for improvements. Clin. Pharmacol. Ther. 107 (5), 1240–1255. 10.1002/cpt.1720 31715018 PMC7232863

[B97] Sinayev A. Peters E. Tusler M. Fraenkel L. (2015). Presenting numeric information with percentages and descriptive risk labels: A randomized trial. Med. Decis. Mak. 35 (8), 937–947. 10.1177/0272989X15584922 PMC4592369 25952743

[B98] Stanek E. .J. Sanders C. .L. Taber K. .A. Khalid M. Patel A. Verbrugge R. .R. (2012). Adoption of pharmacogenomic testing by US physicians: Results of a nationwide survey. Clin. Pharmacol. Ther. 91 (3), 450–458. 10.1038/clpt.2011.306 22278335

[B99] Swen J. .J. van der Wouden C. .H. Manson L. .E. Abdullah-Koolmees H. Blagec K. Blagus T. (2023). A 12-gene pharmacogenetic panel to prevent adverse drug reactions: An open-label, multicentre, controlled, cluster-randomised crossover implementation study. Lancet 401 (10374), 347–356. 10.1016/S0140-6736(22)01841-4 36739136

[B100] Swift C. .G. (2022). Personalised future prescribing using pharmacogenomics: A resume of a joint royal College of physicians/British pharmacological society working party report. Future Healthc. J. 9 (2), 174–178. 10.7861/fhj.rev-9.2.1 35928194 PMC9345247

[B101] The Dutch Pharmacogenetics Working Group (DPWG) (2005). The Dutch pharmacogenetics working group (DPWG). Available at: https://www.knmp.nl/.

[B102] Thornley T. Esquivel B. Wright D. .J. Dop H. .V. .D. Kirkdale C. .L. Youssef E. (2021). Implementation of a pharmacogenomic testing service through community pharmacy in The Netherlands: Results from an early service evaluation. Pharm. (Basel) 9 (1), 38. 10.3390/pharmacy9010038 PMC7930936 33673111

[B103] Thummel K. .E. Lin Y. .S. (2014). Sources of interindividual variability. Methods Mol. Biol. 1113, 363–415. 10.1007/978-1-62703-758-7_17 24523121

[B104] Tsiachristas A. Vallance G. Koleva-Kolarova R. Taylor H. Solomons L. Rizzo G. (2022). Can upfront DPYD extended variant testing reduce toxicity and associated hospital costs of fluoropyrimidine chemotherapy? A propensity score matched analysis of 2022 UK patients. BMC Cancer 22 (1), 458. 10.1186/s12885-022-09576-3 35473510 PMC9044697

[B105] Tuteja S. Salloum R. .G. Elchynski A. .L. Smith D. .M. Rowe E. Blake K. .V. (2022). Multisite evaluation of institutional processes and implementation determinants for pharmacogenetic testing to guide antidepressant therapy. Clin. Transl. Sci. 15 (2), 371–383. 10.1111/cts.13154 34562070 PMC8841452

[B106] United States Food and Drug Administration (2005). Pharmacogenomic data submissions. Available at: https://www.fda.gov/regulatory-information/search-fda-guidance-documents/pharmacogenomic-data-submissions.

[B107] United States Food and Drug Administration (1997). PLAVIX® (clopidogrel bisulfate). Available at: https://www.accessdata.fda.gov/drugsatfda_docs/label/2018/020839s070lbl.pdf.

[B108] United States Food and Drug Administration (2022). Table of pharmacogenetic associations. Available at: https://www.fda.gov/medical-devices/precision-medicine/table-pharmacogenetic-associations.

[B109] United States Food and Drug Administration (2022). Table of pharmacogenomic biomarkers in drug labeling. Available at: https://www.fda.gov/drugs/science-and-research-drugs/table-pharmacogenomic-biomarkers-drug-labeling.

[B110] United States Food and Drug Administration (2009). Tegretol® (carbamazepine). Available at: https://www.accessdata.fda.gov/drugsatfda_docs/label/2009/016608s101,018281s048lbl.pdf.

[B111] US Centers for Medicare & Medicaid Services (2020). MolDX: Pharmacogenomics testing. Local cover. Available at: https://www.cms.gov/medicare-coverage-database/view/lcd.aspx?LCDId=38294&ver=16.

[B112] van der Lee M. Kriek M. Guchelaar H. .J. Swen J. .J. (2020). Technologies for pharmacogenomics: A review. Genes. (Basel). 11 (12), 1456. 10.3390/genes11121456 33291630 PMC7761897

[B113] van der Wouden C. .H. Cambon-Thomsen A. Cecchin E. Cheung K. .C. Davila-Fajardo C. .L. Deneer V. .H. (2017). Implementing pharmacogenomics in Europe: Design and implementation strategy of the ubiquitous pharmacogenomics Consortium. Clin. Pharmacol. Ther. 101 (3), 341–358. 10.1002/cpt.602 28027596

[B114] van der Wouden C. .H. Paasman E. Teichert M. Crone M. .R. Guchelaar H. .J. Swen J. .J. (2020). Assessing the implementation of pharmacogenomic panel-testing in primary care in The Netherlands utilizing a theoretical framework. J. Clin. Med. 9 (3), 814. 10.3390/jcm9030814 32192029 PMC7141350

[B115] van der Wouden C. .H. van Rhenen M. .H. Jama W. .O. .M. Ingelman-Sundberg M. Lauschke V. .M. Konta L. (2019). Development of the PGx-passport: A panel of actionable germline genetic variants for pre-emptive pharmacogenetic testing. Clin. Pharmacol. Ther. 106 (4), 866–873. 10.1002/cpt.1489 31038729 PMC6771671

[B116] Van Driest S. .L. Shi Y. Bowton E. .A. Schildcrout J. .S. Peterson J. .F. Pulley J. (2014). Clinically actionable genotypes among 10,000 patients with preemptive pharmacogenomic testing. Clin. Pharmacol. Ther. 95 (4), 423–431. 10.1038/clpt.2013.229 24253661 PMC3961508

[B117] Varughese L. .A. Bhupathiraju M. Hoffecker G. Terek S. Harr M. Hakonarson H. (2022). Implementing pharmacogenetic testing in gastrointestinal cancers (IMPACT-GI): Study protocol for a pragmatic implementation trial for establishing DPYD and UGT1A1 screening to guide chemotherapy dosing. Front. Oncol. 12, 859846. 10.3389/fonc.2022.859846 35865463 PMC9295185

[B118] Verlouw J. .A. .M. Clemens E. de Vries J. .H. Zolk O. Verkerk A. Am Zehnhoff-Dinnesen A. (2021). A comparison of genotyping arrays. Eur. J. Hum. Genet. 29 (11), 1611–1624. 10.1038/s41431-021-00917-7 34140649 PMC8560858

[B119] Vo T. .T. Bell G. .C. Owusu Obeng A. Hicks J. .K. Dunnenberger H. .M. (2017). Pharmacogenomics implementation: Considerations for selecting a reference laboratory. Pharmacotherapy 37 (9), 1014–1022. 10.1002/phar.1985 28699700

[B120] Vozikis A. Cooper D. .N. Mitropoulou C. Kambouris M. .E. Brand A. Dolzan V. (2016). Test pricing and reimbursement in genomic medicine: Towards a general strategy. Public Health Genomics 19 (6), 352–363. 10.1159/000449152 27676083

[B121] Weitzel K. .W. Duong B. .Q. Arwood M. .J. Owusu-Obeng A. Abul-Husn N. .S. Bernhardt B. .A. (2019). A stepwise approach to implementing pharmacogenetic testing in the primary care setting. Pharmacogenomics 20 (15), 1103–1112. 10.2217/pgs-2019-0053 31588877 PMC6854439

[B122] Whirl-Carrillo M. Huddart R. Gong L. Sangkuhl K. Thorn C. .F. Whaley R. (2021). An evidence-based framework for evaluating pharmacogenomics knowledge for personalized medicine. Clin. Pharmacol. Ther. 110 (3), 563–572. 10.1002/cpt.2350 34216021 PMC8457105

[B123] Whirl-Carrillo M. McDonagh E. .M. Hebert J. .M. Gong L. Sangkuhl K. Thorn C. .F. (2012). Pharmacogenomics knowledge for personalized medicine. Clin. Pharmacol. Ther. 92 (4), 414–417. 10.1038/clpt.2012.96 22992668 PMC3660037

[B124] Williams M. .S. (2019). Early lessons from the implementation of genomic medicine programs. Annu. Rev. Genomics Hum. Genet. 20, 389–411. 10.1146/annurev-genom-083118-014924 30811224

[B125] Wolff N. .D. Wolff J. .A. (2018). A commentary on commercial genetic testing and the future of the genetic counseling profession. J. Genet. Couns. 27 (3), 521–527. 10.1007/s10897-018-0244-6 29524069 PMC5943385

[B126] World Health Organization (WHO) (2021). Essential medicines list. Available at: https://www.who.int/groups/expert-committee-on-selection-and-use-of-essential-medicines/essential-medicines-lists.

[B127] Yang J. .J. Landier W. Yang W. Liu C. Hageman L. Cheng C. (2015). Inherited NUDT15 variant is a genetic determinant of mercaptopurine intolerance in children with acute lymphoblastic leukemia. J. Clin. Oncol. 33 (11), 1235–1242. 10.1200/JCO.2014.59.4671 25624441 PMC4375304

[B128] Zgheib N. .K. Patrinos G. .P. Akika R. Mahfouz R. (2020). Precision medicine in low- and middle-income countries. Clin. Pharmacol. Ther. 107 (1), 29–32. 10.1002/cpt.1649 31674002

